# Early growth response-1 is a regulator of DR5-induced apoptosis in colon cancer cells

**DOI:** 10.1038/sj.bjc.6605545

**Published:** 2010-01-19

**Authors:** D Mahalingam, A Natoni, M Keane, A Samali, E Szegezdi

**Affiliations:** 1 Cell Stress and Apoptosis Research Group, Department of Biochemistry and National Centre of Biomedical Engineering Science, National University of Ireland, Galway, Ireland; 2 Cancer Therapy and Research Center, University of Texas Health Science Center San Antonio, San Antonio, TX, USA; 3 University College Hospital, Galway, Ireland

**Keywords:** tumour necrosis factor-related apoptosis-inducing ligand (TRAIL), early growth response gene-1 (Egr-1), death receptor 5 (DR5), type I extrinsic apoptotic pathway, cellular FLICE inhibitory protein (c-FLIP), colon carcinoma

## Abstract

**Background:**

Tumour necrosis factor-related apoptosis-inducing ligand (TRAIL) induces tumour cell apoptosis by binding to death receptor 4 (DR4) and DR5. DR4 and DR5 activation however can also induce inflammatory and pro-survival signalling. It is not known how these different cellular responses are regulated and what the individual role of DR4 *vs* DR5 is in these processes.

**Methods:**

DNA microarray study was carried out to identify genes differentially expressed after DR4 and DR5 activation. RT–PCR and western blotting was used to examine the expression of early growth response gene-1 (Egr-1) and the proteins of the TRAIL signalling pathway. The function of Egr-1 was studied by siRNA-mediated knockdown and overexpression of a dominant-negative version of Egr-1.

**Results:**

We show that the immediate early gene, Egr-1, regulates TRAIL sensitivity. Egr-1 is constitutively expressed in colon cancer cells and further induced upon activation of DR4 or DR5. Our results also show that DR4 mediates a type II, mitochondrion-dependent apoptotic pathway, whereas DR5 induces a mitochondrion-independent, type I apoptosis in HCT15 colon carcinoma cells. Egr-1 drives c-FLIP expression and the short splice variant of c-FLIP (c-FLIP_S_) specifically inhibits DR5 activation.

**Conclusion:**

Selective knockdown of c-FLIP_S_ sensitises cells to DR5-induced but not DR4-induced apoptosis and Egr-1 exerts an effect as an inhibitor of the DR5-induced apoptotic pathway, possibly by regulating the expression of c-FLIP_S_.

Tumour necrosis factor-related apoptosis-inducing ligand (TRAIL) is a member of the TNF ligand superfamily (Ashkenazi and Dixit, 1998). It induces the extrinsic apoptotic pathway upon binding to its death domain (DD)-containing receptors, TRAIL receptor 1 (death receptor 4 (DR4)) and 2 (death receptor 5 (DR5)). Binding of TRAIL to DR4 and DR5 induces receptor oligomerisation, intracellular DD clustering and recruitment of the adaptor molecule Fas-associated death domain (FADD). The death effector domains (DEDs) of FADD then interact with the DED of pro-caspases 8 and 10, leading to the formation of the death-inducing signaling complex (DISC). The DISC serves as a platform to oligomerise and activate pro-caspases 8 and 10 (Kischkel *et al*, 2000; Sprick *et al*, 2000). Active caspases 8 and 10 are released from the DISC and activate executioner caspases, caspases 3, 6 and 7, committing the cell to death.

Active caspases 8 and 10 can also cleave and activate Bid, a BH3-only member of the Bcl-2 protein family. Truncated Bid then activates Bax and Bak to induce mitochondrial outer membrane permeabilisation and cytochrome *c* release (Eskes *et al*, 2000; Green and Kroemer, 2004). In the cytosol, cytochrome *c* binds to the WD40 domains of the adaptor protein, Apaf-1, which initiates the assembly of the heptameric apoptosome complex. Pro-caspase-9 is recruited to the apoptosome and becomes activated (Green, 2000). Activation of the intrinsic apoptosis pathway in this manner serves to amplify the apoptotic signal and guarantees that the programme is irreversible.

In certain cells, which are classified as type I cells, the intrinsic apoptosis pathway is not required to commit the cell to apoptosis upon TRAIL receptor activation; however, in other cells, which are classified as type II cells, this amplification loop is essential. Overexpression of anti-apoptotic Bcl-2 proteins inhibits TRAIL-induced apoptosis in type II cells only (Fulda *et al*, 2002). Poor activation of pro-caspases 8 and 10 at the DISC is probably one of the major factors that account for the type II phenotype (Scaffidi *et al*, 1999). By competing with pro-caspase-8 for binding to FADD and inhibiting caspase-8 at the DISC, FLICE-inhibitory protein (c-FLIP) may be a key determinant of the type I *vs* type II phenotype (Scaffidi *et al*, 1999; Barnhart *et al*, 2003).

Despite the high homology between DR4 and DR5 and the identical core DISC components recruited to DR4 and DR5, the two receptors are not equally involved in transducing the TRAIL-apoptotic signal (Ichikawa *et al*, 2001; Ashkenazi, 2002; Kelley *et al*, 2005; van der Sloot *et al*, 2006). In the colon cancer cell line, Colo205, we have shown that TRAIL induces apoptosis predominantly through DR5 (van der Sloot *et al*, 2006). Conversely, in the leukaemia cell lines, ML-1 and EM-2, DR4 is the predominant transducer of apoptosis (van Geelen *et al*, 2003; MacFarlane *et al*, 2005). So far, there is no clear explanation for the differential activity of DR4 and DR5. Two reports shed some light to possible, selective regulation of DR4 and DR5. These studies have shown that DcR2 selectively inhibited DR5, but not DR4, through a ligand-dependent or ligand-independent association with DR5 (Clancy *et al*, 2005; Merino *et al*, 2006). But what regulates DR4 function, or whether there are intracellular regulators specific to DR4 or DR5, is completely unknown.

In addition to caspase activation, DR4 and DR5 can activate the transcription factors nuclear factor-*κ* B (NF-*κ*B) and c-Jun, or protein kinases, such as Akt, mitogen-activated protein kinases (MAPKs) such as c-Jun NH2-terminal kinase (JNK) and extracellular signal-regulated kinase (ERK) (Falschlehner *et al*, 2007), which lead to the activation of multiple signal transduction pathways. To examine the role of these pathways in the regulation of DR4 or DR5 function, genes induced or repressed by recombinant human TRAIL (rhTRAIL) and DR5-selective rhTRAIL variants were determined in a colon cancer cell model using cDNA microarray technology. In this report we show that the immediate early gene, Egr-1, is constitutively expressed in colon cancer cells and further induced in response to rhTRAIL by both DR4 and DR5. Furthermore, we show that the short isoform of c-FLIP controls the activity of the DR5 receptor, but not of DR4. The constitutively expressed Egr-1 inhibits TRAIL-mediated apoptosis, probably by driving constitutive c-FLIP expression.

## Materials and methods

### Cell culture and treatments

Colo205 cells were obtained from American Tissue Culture Collection (ATCC). HCT15 and HCA7 cells were a kind gift from Professor L Egan (National University of Ireland, Galway). Colo205 and HCT15 cells were maintained in RPMI-1640 medium and HCA7 in DMEM medium, both media supplemented with 10% fetal bovine serum (FBS), 2 mM glutamine, 50 U ml^–1^ penicillin and 50 mg ml^–1^ streptomycin at 37 °C, 5% CO_2_ in a humidified incubator. Cells were seeded at 2 × 10^5^ cells ml^–1^ at 1 day before treatment. To induce apoptosis, cells were treated with rhTRAIL (non-tagged, fragment of amino acids 114–281, Triskel Therapeutics, Groningen, The Netherlands) DR5-selective mutants D269H, D269H/E195R, agonistic DR4 or DR5 antibodies (Novartis Pharmaceuticals, Basel, Switzerland), recombinant human TNF (PromoCell, Heidelberg, Germany) or agonistic anti-Fas antibody (clone CH-11, MBL International, Woburn, MA, USA) at the concentration and times specified in the figure legends. All reagents were from Sigma-Aldrich (St Louis, MD, USA) unless otherwise stated.

### Cell viability assay

Cell viability was monitored using 3-(4, 5-dimethylthiazolyl)-2, 5-diphenyl tetrazolium bromide (MTT) assay. After treatment, MTT (0.5 mg ml^–1^) was added to cells and incubated for 3 h at 37 °C. The reaction was stopped by addition of an MTT stop solution of 20% SDS in 50% dimethyl formamide. The purple formazan precipitate generated was allowed to dissolve for 1 h on an orbital shaker. The colour intensity was measured at 550 nm on a Wallac Victor 1420 Multilabel counter (PerkinElmer Life Sciences, Waltham, MA, USA). Cell viability was expressed relative to the absorbance of untreated cells, which was taken as 100% viable.

### Cell death assay

Cell death was monitored by labelling of phosphatidyl serine externalised on the surface of apoptotic cells with Annexin-V-FITC (IQ Corporation, Groningen, The Netherlands) or by haematoxylin and eosin staining of cytospins. For Annexin V staining, cells were collected by gentle trypsinisation and incubated for 10 min at 37 °C to allow membrane recovery. Cells were pelleted by centrifugation at 350 **g** and incubated with Annexin-V-FITC in calcium buffer (10 mM HEPES/NaOH, pH 7.4, 140 mM NaCl and 2.5 mM CaCl_2_) for 15 min on ice in the dark. Cells were washed in calcium buffer before acquisition on a FacsCalibur flow cytometer (Becton Dickinson, Franklin Lakes, NJ, USA). Analysis was performed using Cell Quest software (Becton Dickinson). Haematoxyin–eosin staining has been carried out as described before (Szegezdi *et al*, 2008).

### Microarray analysis

Global gene expression analysis was carried out on RNA prepared from Colo205 cells exposed to rhTRAIL, D269H or D269H/E195R for 1 h. Microarray hybridisation and bioinformatics analysis was performed by ArraDx Array-Based Diagnostics using Affymetrix human HgU133 Plus 2.0 gene chips in triplicate (Belfast, UK). Single-channel experiments were carried out with all RNA samples labelled with biotin. In brief, double-stranded cDNA was synthesised from 5 *μ*g total RNA, and purified and biotin labelled. Labelled cRNA was fragmented, purified and quantified before its hybridisation to the gene chips for 16 h at 45 °C. The arrays were washed, stained with streptavidin-phycoerythrin solution for 10 min at 25 °C, and then re-washed and probed with a biotinylated antibody solution for 10 min at 25 °C. The streptavidin-phycoerythrin solution was added for a further 10 min and washed before scanning. The GeneSpring data analysis program (Silicon Genetics/Agilent, Santa Clara, CA, USA) was used for bioinformatical analysis. Fold increases or decreases induced were compiled for the treatments. Genes with greater than a two-fold change and a *t*-test *P*-value of <0.05 were considered differentially regulated.

### RNA isolation and RT–PCR

Total RNA was isolated using GenElute RNA miniprep kit (Sigma-Aldrich) according to the manufacturer's protocol. Reverse transcription (RT) was carried out with 2 *μ*g RNA using oligo (dT) primers (Invitrogen, Carlsbad, CA, USA) and AMV reverse transcriptase. The cDNA product was subjected to 25–30 cycles of PCR using primers specific for Egr-1, c-Jun, TEA domain family member 1 (TEAD-1), naked cuticle homologue 2 (NKD2), voltage-dependent anion channel 3 (VDAC3), NF-*κ*B inhibitor-*α*/inhibitor-*κ* B-*α* (NFKBIA/I*κ*B*α*) and NF-*κ* light polypeptide gene enhancer in B-cells inhibitor-*ζ* (NFKBIZ/I*κ*B*ζ*). For normalisation, GAPDH PCR was carried out. The primers used for the PCR reactions are listed below.


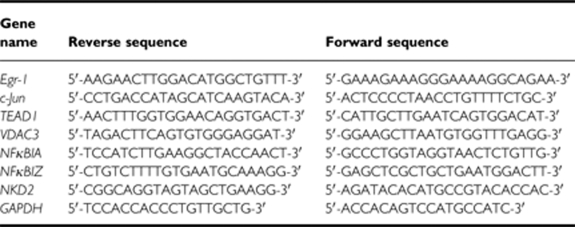


### Protein lysate preparation and western blot analysis

Cells were lysed in buffer containing 100 mM Tris–HCl, pH 8.0, 1% Triton X-100, 200 mM sodium chloride (NaCl), 5 mM EDTA, 10% glycerol, 1 mM dithiothreitol (DTT), 1 mM phenylmethylsulphonyl fluoride (PMSF), 5 *μ*g ml^–1^ aprotinin, 2.5 *μ*g ml^–1^ leupeptin, 1 mM sodium orthovanadate (Na_2_VO_3_) and 1 mM sodium fluoride (NaF). Cellular proteins (30 *μ*g) were separated by electrophoresis on 8–10% SDS polyacrylamide gels and transferred onto nitrocellulose membranes. After blocking in 5% non-fat milk and 0.05% Tween-20 in PBS, blots were incubated with rabbit antibodies to total Egr-1 (1 : 1000 dilution, Santa Cruz Biotechnologies, Santa Cruz, CA, USA), Mcl-1, Bax (1 : 1000, Cell Signaling Technology, Danvers, MA, USA) or mouse monoclonal antibodies to c-FLIP (1 : 500, Alexis Pharmaceuticals, Axxora UK Ltd., Nottingham, UK), Bcl-X_L_ (clone H-5, 1 : 200, Santa Cruz Biotechnologies), Bcl-2 (clone 100, 1 : 1000, Santa Cruz Biotechnologies) or X-linked inhibitor of apoptosis protein (XIAP; 1 : 2000, Assay Designs, Ann Arbon, MI, USA). For detection, appropriate horseradish peroxidase-conjugated goat secondary antibodies were used (Thermo Fisher Scientific, Rockford, IL, USA). Protein bands were visualised with SuperSignal West Pico Chemiluminescent Substrate (Pierce) on X-ray film (Agfa, Morstel, Belgium).

### Transfections and plasmids

Dominant-negative Egr-1 construct (EBGN-EGR-1) expresses a truncated version of murine Egr-1 lacking the transactivational domain and containing only the zinc-finger DNA-binding site (amino acids 322–533) fused to GST. The empty vector, EBGN, contains a nuclear-expressed GST (Al-Sarraj *et al*, 2005); both these vectors are a kind gift from Professor G Thiel (University of Saarland Medical Center, Homburg, Germany). pEBS1^4^luc, an Egr-1 reporter construct, contains four copies of Egr-1 response element of the Egr-1 gene promoter in the pGL3-promoter vector (also a gift from Professor G Thiel, University of Saarland Medical Center) (Al-Sarraj *et al*, 2005). To normalise for transfection efficiency, a constitutive Renilla luciferase expressing plasmid was used (pRL-CMV, Promega Corporation, Madison, WI, USA). For transfection, HCT15 cells (2 × 10^6^) were pelleted and resuspended in transfection solution V (Lonza Group Ltd., Basel, Switzerland) containing 2.5 *μ*g of plasmid unless otherwise stated. Transfection was performed by nucleofection using program T13 according to the manufacturer's protocol (Amaxa). GFP plasmid (2.5 *μ*g) was used to determine transfection efficiency, which was 48±7%. Control cells were subjected to the same transfection condition without any plasmids. At 24 h after transfection, cells were resuspended in media and seeded for Annexin V and protein assays. Similarly, stable transfection of Bcl-2 or empty vector (Neo) was carried out in HCT15 cells using the same transfection protocol (a kind gift from Dr Peter Daniel, University of Berlin, Berlin, Germany). Pools of stable clones were selected with 1 *μ*M of G418. siRNA transfection was carried out by the same nucleofection protocol as for plasmids using 50–75 nM siRNA. The following c-FLIP sequences were targeted: c-FLIP_S/L1_: 5′-GGAGCAGGGACAAGTTACA-3′, c-FLIP_S/L2_: 5′-GCAAGGAGAAGAGTTTCTT-3′, c-FLIP_S/L3_: 5′-GAGGTAAGCTGTCTGTCGG-3′ (Nakajima *et al*, 2008), c-FLIP_S1_: 5′-CACCCTATGCCCATTGTCC-3′, cFLIP_S2_: 5′-CATGGAACTGCCTCTACTT-3′ (Zhang *et al*, 2004; Longley *et al*, 2006). The GFP target sequence was: 5′-GGCUACGUCCAGGAGCGCACC-3′. To knock down Egr-1, an siRNA Smartpool containing a mixture of four Egr-1-specific siRNAs was used (Dharmacon, Thermo Fisher Scientific, Rockford, IL, USA). Transfection was carried out as for c-FLIP siRNAs.

### Luciferase assay

Luciferase activity was determined using the Dual Glo Luciferase assay system (Promega). The measurement was carried out according to the manufacturer's instructions.

### Cell surface expression of TRAIL receptors

Cells were washed twice in PBS containing 1% BSA and then incubated with monoclonal antibodies to DR4 or DR5 (Alexis) for 40 min. After two wash steps with PBS–BSA, anti-mouse IgG-FITC (Sigma) secondary antibody was added for 30 min. All incubations were carried out on ice. Negative controls contained isotype control antibody. Cells were analysed using FacsCalibur flow cytometer.

### Statistical analysis

Differences in Annexin V staining between the treatment groups were analysed using a non-paired Student's *t*-test, with a significance level of *P*<0.05. Error bars shown are s.e.m. All statistical analyses were performed using Graphpad Prism 4 (GraphPad Softward Inc, San Diego, CA, USA).

## Results

### Colon carcinoma cells are sensitive to rhTRAIL but use different receptors to transmit the death signal

**Figure 1:**
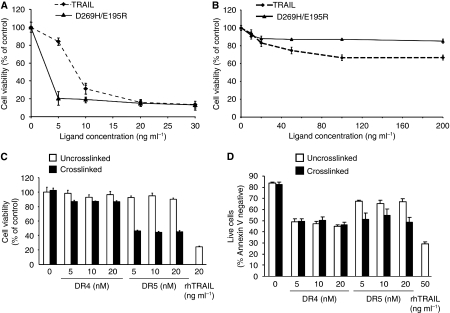
Colon carcinoma cells are sensitive to rhTRAIL with Colo205 cells responding to DR5 stimulation and HCT15 to both DR4 and DR5. Cell viability of Colo205 (**A**) and HCT15 (**B**) cells treated with WT rhTRAIL and DR5-selective TRAIL variant D269H/E195R (5–30 and 10–200 ng ml^–1^, respectively) for 24 h. Cell viability was measured using MTT assay. Values are expressed as a percentage of untreated cells and presented as mean±s.e.m. of three independent experiments. Colo205 (**C**) and HCT15 (**D**) cells were treated with 5–20 nM of agonistic DR4 (DR4) and DR5 antibodies (DR5) for 24 and 5 h, respectively. Where indicated, agonistic antibodies were crosslinked using 15–60 nM of crosslinking antibody for 30 min before cell treatment. Cell death was measured using MTT assay in Colo205 cells and Annexin V staining in HCT15 cells. The results are presented as mean±s.e.m. of three independent experiments.

To determine the sensitivity of colon carcinomas to TRAIL-induced apoptosis, Colo205 and HCT15 cell lines were treated with increasing concentrations of rhTRAIL or DR5-selective TRAIL variant, D269H/E195R for 3 h (Figure 1A) (van der Sloot *et al*, 2006). Colo205 cells were more sensitive to D269H/E195R than rhTRAIL, whereas in HCT15 cells rhTRAIL seemed to be a stronger inducer of death (Figure 1A and B).

To determine what TRAIL receptors transmitted the apoptotic signal, cells were treated with agonistic DR4- and DR5-selective antibodies (Novartis) for 3 and 5 h, for Colo205 and HCT15 cells, respectively. In the absence of crosslinking of anti-DR4 and anti-DR5 with a secondary antibody, the agonistic antibodies induced similar, low level of apoptosis in Colo205 cells. To more closely mimic the action of the trimeric TRAIL ligand on the receptors, the agonistic antibodies were crosslinked with a secondary antibody through their Fc regions. Crosslinking is likely to enhance clustering and thus the activation of the death receptors in a similar manner as it has recently been shown for Fas (Scott *et al*, 2009). Crosslinking significantly increased the activity of the DR5-agonistic antibody, but not of the DR4 antibody (Figure 1C), agreeing with previous reports showing that DR5, but not DR4, requires crosslinking for optimal activation (Kelley *et al*, 2005). In HCT15 cells, both the DR4 and DR5 antibodies induced apoptosis, with the DR4 antibody being a stronger death inducer. Again, enhanced apoptosis was observed after crosslinking of the DR5, but not the DR4-agonistic antibody (Figure 1D). These results show that in Colo205 cells, TRAIL signals apoptosis primarily through the DR5 receptor, whereas in HCT15 cells, the TRAIL death signal can be transmitted by both receptors.

### rhTRAIL induces Egr-1 through both DR4 and DR5

To generate a profile of early response genes induced by TRAIL receptor activation, Colo205 cells were treated with either rhTRAIL or DR5-selective rhTRAIL variants (D269H and D269H/E195R) for 1 h. Microarray analysis was carried out on Affymetrix human HgU133 Plus 2.0 GeneChips in triplicate. The concentration of TRAIL and DR5 variants was chosen to be 10 ng ml^–1^ as it induced near-maximal apoptosis in Colo205 cells (Figure 1A). By examining the temporal induction of TRAIL-regulated genes known from the literature, such as BTG family 3 (BTG3), ubiquitin-specific protease 24 (USP24), KIAA0770 and cyclin T1 (CCNT1) upregulated by TRAIL and non-POU-domain containing octamer binding (NoNo), downregulated by TRAIL) (Kumar-Sinha *et al*, 2002), it was determined that gene expressional changes are detectable from 1 h of TRAIL treatment and thus this time point was chosen for the microarray analysis (Supplementary Figure 1).

**Figure 2:**
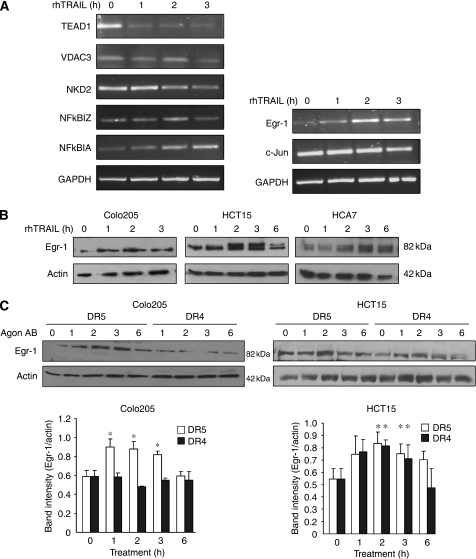
rhTRAIL induces Egr-1 expression that can be mediated by both DR4 and DR5. (**A**) Validation of cDNA microarray results. Colo205 cells were treated with 10 ng ml^–1^ of WT rhTRAIL and total RNA was isolated at the times indicated. mRNA expression of TEAD1, VDAC3, NKD2, Egr-1, c-Jun, NFKBIA and NFKBIZ were assessed using RT–PCR. GAPDH was used as internal control. The figure shows a representative image of three independent experiments. (**B**) Induction of Egr-1 protein by TRAIL receptor activation. Colo205, HCT15 and HCA7 cells were treated with rhTRAIL at a concentration of 10 ng ml^–1^ (Colo205) and 50 ng ml^–1^ (HCT15 and HCA7). Cell lysates were prepared at the indicated times and analysed for the expression of Egr-1 using western blotting. Actin expression was detected for loading control. The figure shows representative images of two independent experiments. (**C**) The role of DR4 and DR5 in Egr-1 induction. Colo205 and HCT15 cells were treated with 10 nM of crosslinked agonistic DR4 (DR4) and DR5 antibodies (DR5). Cells lysates were prepared at the indicated times and expression of Egr-1 protein was analysed using western blotting. Detection of actin was used as a loading control. Densitometric quantification of Egr-1 levels. The graph shows averaged Egr-1 band densities normalised for total *β*-actin levels in the lysates. Results are presented as means±s.e.m. of three independent experiments; ^*^P<0.05 comparing Egr-1 induction at each time points to control.

The microarray analysis revealed 69 genes differentially expressed in response to at least one treatment. Cluster analysis identified four genes regulated by both TRAIL and DR5-selective variants. These were CDC42 effector protein 1 (CDC42EP1), Egr-1, TEAD1 and VDAC3. Functional clustering identified that the regulated genes have a role in intracellular transport, cellular proliferation, post-translational modification and transcription–translation regulation (Table 1A). Of these genes, seven candidates were selected for further analysis based on proposed biological function and fold induction–repression by rhTRAIL (Table 1B). The full list of genes differentially expressed can be found in Supplementary Table 1. Except the induction of c-Jun, upregulation of Egr-1, NFKBIA/I*κ*B*α* and NFKBIZ/I*κ*B*ζ* and downregulation of Homo sapiens NKD2, VDAC3 and TEAD1 in Colo205 cells by rhTRAIL were all confirmed validating the microarray results (Figure 2A).

Egr-1, which is also known as NGFI-A, zif268, krox24 and Tis8, is a transcription factor implicated in tumour progression and apoptosis after diverse stimuli (Thiel and Cibelli, 2002). Currently, there is no information about its role in TRAIL-induced apoptosis. Analysis of Egr-1 protein expression in colon carcinoma cell lines (Colo205, HCT15 and HCA7) showed high basal expression of Egr-1 and its further induction in response to rhTRAIL, DR4- and DR5-agonistic antibodies (Figure 2B and C). A double band of Egr-1 was detected in HCT15 and HCA7 cells. The upper band probably corresponds to a phosphorylated form of Egr-1, which has been shown to increase its activity (Beckmann and Wilce, 1997). For quantification, blots were also probed for *β*-actin and the densitometric ratio of Egr-1 to *β*-actin was calculated (Figure 2C).

### Overexpression of dominant-negative Egr-1 potentiates apoptosis induction by DR5

**Figure 3:**
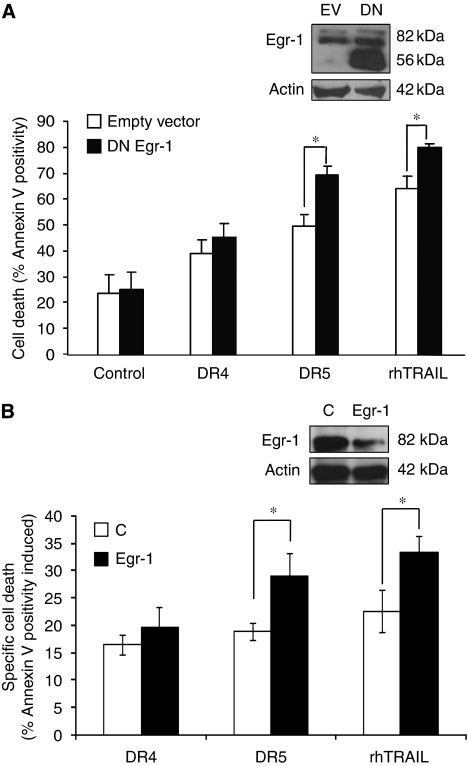
Inhibition or knockdown of Egr-1 potentiates rhTRAIL- and DR5-induced apoptosis. (**A**) Effect of dominant-negative Egr-1 (DN-Egr-1) expression on TRAIL-, DR4- and DR5-induced apoptosis in HCT15 cells. HCT15 cells were transiently transfected with EBGN-Egr-1 (DN) or empty vector (EV). Cell lysates of parental cells (C), EV- and DN-Egr-1-transfected cells were analysed for overexpression of DN-Egr-1 at 24 h after transfection using western blotting (inlet). HCT15 cells transfected with pEBGN-EGR-1 (DN-Egr-1) were treated at 24 h after transfection with either 10 nM of crosslinked agonistic DR4/DR5 antibody or 50 ng ml^–1^ rhTRAIL for 5 h and apoptosis was assessed using Annexin V staining (DR4, DR5 and rhTRAIL). (**B**) Knockdown of Egr-1 sensitises HCT15 cells to TRAIL and DR5-induced apoptosis. HCT15 cells were transiently transfected with a Smartpool siRNA mix against Egr-1 (Egr-1) or scrambled siRNA (control siRNA, C). Knockdown of Egr-1 was confirmed 24 h after transfection using Western blotting (inlet). Cells were treated as described in point (**A**) and induction of apoptosis was measured with Annexin V staining. Results are presented as means±s.e.m. of at least three independent experiments; ^*^Significant difference with *P*<0.05.

To determine whether Egr-1 has any role in TRAIL-induced apoptosis, HCT15 cells were transiently transfected with a plasmid expressing dominant-negative Egr-1 (EBGN-Egr-1) that contains only the DNA-binding domain of Egr-1 fused to GST (Al-Sarraj *et al*, 2005). Overexpression of dominant-negative Egr-1 protein (DN-Egr-1) was confirmed by western blot analysis using Egr-1 antibody (inlet, Figure 3A). On the blot, the lower (approximately 56 kDa) band represents the truncated, DN-Egr-1. To inhibit Egr-1 activity, 2.5 *μ*g of DN-Egr-1 plasmid was transfected into the cells, as this amount was found to fully block Egr-1 transcriptional activity for at least 48 h after transfection (Supplementary Figure 2A). After 5 h treatment with 10 nM agonistic DR5 antibody or rhTRAIL, HCT15 cells overexpressing DN-Egr-1 suffered significantly more apoptosis than untransfected cells or cells transfected with the empty vector (Figure 3A). Interestingly, no enhancement in apoptosis was observed in cells treated with agonistic DR4 antibody (Figure 3A). Knockdown of Egr-1 with siRNA (Smartpool, Dharmacon) also increased the sensitivity of HCT15 cells to DR5 activation, but not to DR4 activation (Figure 3B).

### Only DR4-induced, but not DR5-induced, apoptosis requires mitochondrial amplification in HCT15 cells

**Figure 4:**
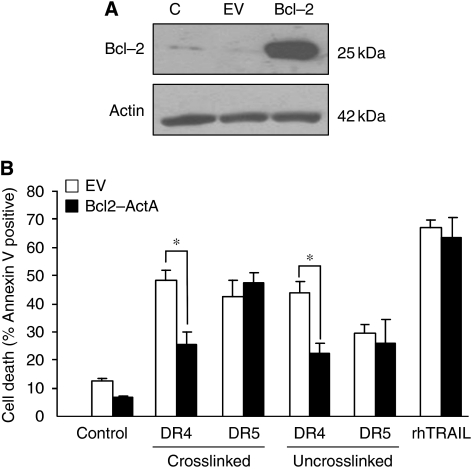
Only DR4-, but not DR5-mediated, apoptosis requires mitochondrial amplification in HCT15 cells. (**A**) Overexpression of mitochondrial-localised Bcl-2. HCT15 cells were stably transfected with mitochondrial-localised Bcl-2 (Bcl-2-ActA: Bcl-2) expressing plasmid or empty vector (EV). Overexpression of Blcl-2-ActA was confirmed using western blotting. (**B**) Bcl-2 ActA protects HCT15 cells from DR4-, but not from DR5- or rhTRAIL-induced apoptosis. Cells were treated with 10 nM of crosslinked or uncrosslinked agonistic DR4 and DR5 antibodies or 50 ng ml^–1^ of rhTRAIL for 12 h and apoptosis was measured using Annexin V staining (DR4, DR5, rhTRAIL labels). Results are presented as means±s.e.m. of three independent experiments. Asterisks (^*^) designate significant difference (*P*<0.05) between the indicated sample pairs.

As overexpression of DN-Egr-1 affected only the DR5-mediated but not the DR4-mediated apoptotic pathway, we wanted to determine whether DR5 and DR4 signal apoptosis through the same pathway in HCT15 cells. As Egr-1 has been reported to regulate the expression of Bcl-2 proteins (Huang *et al*, 1998b; Ahmed, 2004), first the requirement for mitochondrial amplification for DR4- and DR5-mediated apoptosis was assessed. To this end, stable, mitochondrial-targeted Bcl-2 overexpressing HCT15 cells were generated (mass pool of stable transfectants of Bcl-2-ActA overexpressing cells; Figure 4A) and treated with agonistic DR4 and DR5 antibodies (10 nM) or rhTRAIL (50 ng ml^–1^). Cells were treated for 12 h to allow all cells affected to undergo apoptosis. Bcl-2 overexpression reduced the level of apoptosis induced by DR4, but not by DR5 or rhTRAIL (Figure 4B), indicating that in HCT15 cells the DR4-induced apoptotic pathway requires mitochondrial amplification, whereas the DR5-induced pathway does not. The effect of DN-Egr-1 on the expression of key mitochondrial proteins was nonetheless examined. Western blot analysis showed that overexpression of DN-EGR-1 did not alter the expression levels of Bax, Bcl-2, Bcl-X_L_, Mcl-1 or XIAP (Supplementary Figure 2B).

### DN-Egr-1 overexpression reduces c-FLIP expression in HCT15 cells

**Figure 5:**
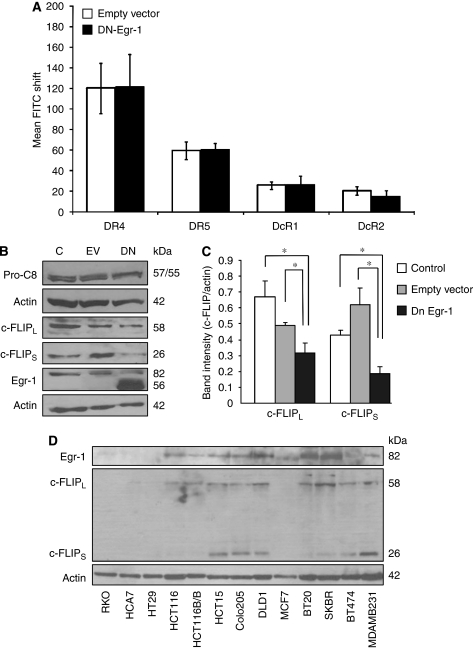
DN-Egr-1 reduces c-FLIP expression in HCT15 cells. (**A**) Effect of DN-Egr-1 on the cell surface expression of TRAIL receptors. Mock- (EV) or DN-Egr-1 (DN)-transfected HCT15 cells were analysed for surface expression of DR4, DR5, DcR1 and DcR2 at 48 h after transfection using flow cytometry. The graph shows the averaged geometric mean of histogram peaks corrected with isotype control of five independent experiments. (**B**) Effect of DN-Egr-1 on pro-caspase-8 and c-FLIP expression. Lysates of untransfected (**C**), mock- (EV) or DN-Egr-1 (DN)-transfected HCT15 cells at 48 h after transfection were analysed for pro-caspase-8 protein expression using western blotting. The image represents two independent experiments. (**C**) Densitometric quantification of c-FLIP_L_ and c-FLIP_S_ blots using GeneTools software (version 3.07, SynGene, Cambridge, UK). c-FLIP expression values were normalised to actin expression level. Results are presented as mean±s.e.m. of three independent experiments, ^*^P<0.05. (**D**) Expression of Egr-1 and c-FLIP in colon and breast cancer cell lines. Cell lysates of untreated cells were prepared and analysed for the expression of Egr-1, c-FLIP_L_, c-FLIP_S_ and *β*-actin using western blotting. The image is representative of two independent experiments.

As the mitochondrial pathway is not required for DR5-mediated apoptosis in HCT15 cells, we next examined whether overexpression of DN-Egr-1 can modulate the expression of the components of the TRAIL-DISC: TRAIL receptors, pro-caspase-8 and c-FLIP. DN-Egr-1 did not have any effect on the surface expression of any of the four TRAIL receptors or the expression of pro-caspase-8 (Figure 5A and B). On the other hand, overexpression of DN-Egr-1 decreased the expression of c-FLIP, especially of the short c-FLIP isoform (c-FLIP_S_, Figure 5B and C). Knockdown of Egr-1 also reduced the expression of c-FLIP, and the reduction was more pronounced in the short c-FLIP splice variant (Supplementary Figure 3A and B). When the expression of Egr-1 and c-FLIP was studied in colon and breast cancer cell lines, we found that high Egr-1 expression often associates with high c-FLIP expression, especially c-FLIP_S_ (Figure 5D). As c-FLIP also inhibits death signalling through the TNF receptor and Fas, the effect of DN-Egr-1 on TNF and Fas sensitivity of HCT15 cells was examined. We found that DN-Egr-1 increased apoptosis induced by both TNF and agonistic anti-Fas antibody (Supplementary Figure 3C).

By analysing the 5′ region of the human *c-FLIP* gene using the Transcription Element Search System web interface (Schug, 2008) (TESS, http://www.cbil.upenn.edu/cgi-bin/tess/tess?RQ=WELCOME), we found the 9 nucleotide Egr-1 binding site (GSG motif: CGGGGGCG) at the beginning of the first intron (Supplementary Figure 4). The binding sequence has a nearly 100% identity to the weighted matrix consensus sequence (Swirnoff and Milbrandt, 1995) (http://www.cbil.upenn.edu/cgi-bin/tess/tess?request=IMD-DBRTRV-Accno&key=I00117), indicating that it is a high-affinity site for Egr-1 binding.

### Selective downregulation of c-FLIP_S_ enhances DR5, but not DR4-induced apoptosis in HCT5 cells

**Figure 6:**
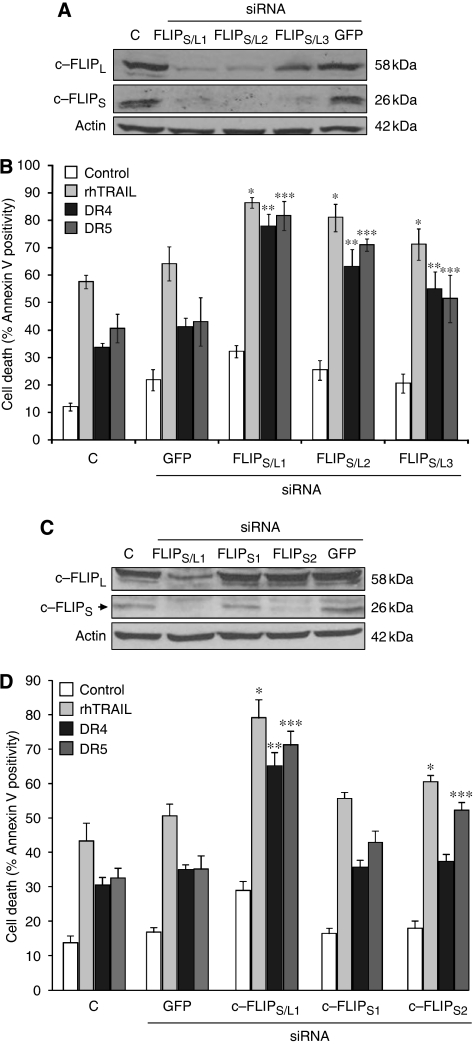
Knockdown of c-FLIP_S_ potentiates DR5-induced apoptosis in HCT5 cells. (**A**) Cell lysates were prepared from HCT15 cells transfected with three different siRNA constructs targeting the common region of c-FLIP_S_ and c-FLIP_L_ (c-FLIP_S/L1−3_) or GFP as a negative control at 24 h after transfection and knockdown of c-FLIP_L_ and c-FLIP_S_ were measured using western blot analysis. Actin expression was determined to serve as loading control. The image is representative of three independent experiments. (**B**) Simultaneous knockdown of c-FLIP_S_ and c-FLIP_L_ potentiates TRAIL, DR4- and DR5-induced HCT15 apoptosis. HCT15 cells transfected as in (**A**) were treated at 24 h after transfection with 50 ng ml^–1^ rhTRAIL or 10 nM crosslinked agonistic DR4 (DR4) and DR5 (DR5) antibodies for 5 h and apoptosis was assessed using Annexin V staining. Results are presented as means±s.e.m. of three independent experiments; ^*^P<0.05. (**C**) Selective knockdown of c-FLIP_S_. Cell lysates were prepared from HCT15 cells transfected with two different siRNA constructs targeting the exon 7 in c-FLIP_S_ (c-FLIP_S1, 2_) or GFP as a negative control at 24 h after transfection and knockdown of c-FLIP_L_ and c-FLIP_S_ were measured using western blot analysis. Actin expression was determined to serve as loading control. The image is representative of three independent experiments. (**D**) Selective knockdown of c-FLIP_S_ potentiates DR5-, but not DR4-induced HCT15 apoptosis. HCT15 cells transfected as in (**C**) were treated as in (**B**) and apoptosis was assessed using Annexin V staining. Results are presented as means±s.e.m. of three independent experiments; ^*^P<0.05. Asterisks (^*^) label samples with significant difference from the TRAIL-treated (^*^), DR4-treated (^**^) or DR5-treated (^***^) GFP-transfected sample (*P*<0.05).

siRNA oligonucletides targeting three regions of c-FLIP, common in c-FLIP_L_ and c-FLIP_S_ (c-FLIP_L/S1–3_) were designed and transfected into HCT15 cells. Downregulation of c-FLIP_L_ and c-FLIP_S_ was confirmed using western blot analysis at 24 h after transfection (Figure 6A). The c-FLIP_L/S_ siRNA resulted in downregulation of both c-FLIP_L_ and c-FLIP_S_._._ HCT15 cells transfected with the siRNAs were treated with 50 ng ml^–1^ rhTRAIL, 10 nM crosslinked DR4 or DR5 antibodies for 5 h and induction of apoptosis was assessed. All treatments resulted in enhanced cell death in c-FLIP_L/S_ siRNA-transfected cells when compared with non-transfected or GFP siRNA-transfected cells (Figure 6B). In view of the greater downregulation of c-FLIPs than c-FLIP_L_ by DN Egr-1, we chose to specifically downregulate c-FLIPs. The only unique region of c-FLIP_S_ in comparison to c-FLIP_L_ is the short exon 7 (Golks *et al*, 2005), which contained only two stretches of sequences targetable with siRNA. Of these two siRNAs, however, only one (c-FLIP_S-2_) was able to significantly downregulate c-FLIP_S_ expression, the c-FLIP_S_ siRNA targeting the first region (c-FLIP_S-1_) seemed to be ineffective (Figure 6C). c-FLIP_S_ siRNA-transfected HCT15 cells were treated with WT rhTRAIL, DR4- or DR5-agonistic antibodies and the apoptosis-potentiating effect of c-FLIP_S_ knockdown was measured. c-FLIP_S-1_ did not enhance cell death in response to any of the treatments, as expected. However, c-FLIP_S-2_ siRNA-transfected cells showed increased cell death in response to WT rhTRAIL and DR5 antibody, but not to DR4 antibody; that is, c-FLIP_S_ knockdown mirrored the effect of DN Egr-1 (Figure 6D).

## Discussion

Death ligands induce apoptosis in tumour cells (Ashkenazi and Dixit, 1998; Papenfuss *et al*, 2008) independent of p53 and thus offer an alternative therapy to genotoxic agents (Ashkenazi, 2008). Various formulations of DR agonists, TNF, Fas ligand and TRAIL are in phase I and II clinical trials with promising results (Papenfuss *et al*, 2008; Mahalingam *et al*, 2009). Of the death ligands, TRAIL is of special interest, as in contrast to TNF and FasL, it has minimal or no toxic side effects (Ashkenazi *et al*, 2008). However, the regulation of TRAIL-induced apoptosis, the mechanism of TRAIL resistance and the differential role of DR4 and DR5 in TRAIL signalling is not sufficiently understood (Di Pietro and Zauli, 2004; Duiker *et al*, 2006).

To gain insight into the regulation of TRAIL-induced apoptosis, we identified the early response genes regulated by TRAIL receptor activation. Gene ontological clustering identified regulation of gene transcription as one of the main biological functions regulated by TRAIL. Among the TRAIL-regulated transcription factors were TEAD1 and Egr-1. Egr-1 (also known as NGFI-A, zif268, krox24 and Tis8) is a zinc-finger transcriptional factor that belongs to a group of early response genes together with Egr-2, Egr-3, Egr-4, Egr-*α* and the tumour suppressor, Wilms' tumour gene product, WT1. Egr-1 has been implicated in the control of cell growth, survival and transformation (Thiel and Cibelli, 2002; Ahmed, 2004). Egr-1 has also been connected to the development of human cancers. It has been proposed to have a role in multistage carcinogenesis in the skin (Riggs *et al*, 2000). High levels of constitutive Egr-1 expression have been observed in most human prostate cancers and found to correlate with more advanced stages of malignancy and poor prognosis (Eid *et al*, 1998). Moreover, tumour progression in transgenic mouse models of prostate cancer was reported to be significantly impaired when Egr-1 was not expressed (Abdulkadir *et al*, 2001). Egr-1 basal expression was also found to be much higher in gastric cancer tissues than in normal gastric mucosa and high Egr-1 mRNA expression correlated with metastasis to lymph nodes and remote organs (Kobayashi *et al*, 2002).

To date, the studies analysing the functions of Egr-1 have been contradictory, with reports of both cytoprotective and pro-apoptotic functions in tumour cells (Huang *et al*, 1998a; Virolle *et al*, 2001). Egr-1 induction has been implicated as a key event in response to ionising radiation-induced growth arrest or cell death mediated by Egr-1 target genes such as TNF-*α*, p53, Retinoblastoma and Bax (Ahmed, 2004). The role of Egr-1 in TRAIL-induced apoptosis is limited. One study showed that Egr-1 negatively regulates survivin expression and hence sensitises cell lines to TRAIL-induced apoptosis (Wagner *et al*, 2008). Another study linked Egr-1 to TRAIL that showed that TNF and TRAIL are released from irradiated (IR) tumour cells and induce bystander death of neighbouring/IR-unaffected cells. Although TNF secretion was mediated by Egr-1, TRAIL secretion only occurred in a tumour cells line that did not express functional Egr-1 (Shareef *et al*, 2007). This study also indicates that during irradiation or genotoxic drug exposure, Egr-1 enhances tumour regression by inducing a bystander effect.

Our study found that Egr-1 is not only rapidly induced by TRAIL, but is also constitutively expressed at a relatively high level in many colon carcinoma cell lines. Another study also found Egr-1 upregulation at the mRNA level in early-onset colorectal cancers (Hong *et al*, 2007). Inhibition of Egr-1 by overexpressing DN-Egr-1 augmented cell death induced by TRAIL through the DR5, but not through the DR4 receptor. The differential role of DR4 and DR5 may relate to our finding that in HCT15 cells DR4-mediated apoptosis requires mitochondrial amplification whereas DR5 stimulation induces a type I, mitochondrial-independent apoptotic pathway. Inhibition of Egr-1 however did not alter expression of the Bcl-2 family members, Bax, Bcl-2, Bcl-X_L_ or Mcl-1. In addition, other studies examining the regulation of Bcl-2 proteins by Egr-1 have shown induction of the pro-apoptotic member, Bax, and repression of Bcl-2, which would enhance the DR4-mediated, type II pathway, rather than the DR5-mediated type I pathway (Huang *et al*, 1998b; Ahmed, 2004; Zagurovskaya *et al*, 2009).

Inhibition of Egr-1 by a dominant-negative mutant, or siRNA-mediated knockdown, significantly decreased the expression of the caspase-8 inhibitor protein, c-FLIP, especially its short isoform (c-FLIP_S_) and Egr-1 expression associated with high c-FLIP expression in a number of cancer cell lines. The reduction in c-FLIP expression was only partial; however, this experiment probably underestimated the effect of Egr-1 on c-FLIP expression because the maximum transfection efficiency of DN-Egr-1 that we could achieve was 50% in HCT15 cells. Nonetheless, we cannot exclude the contribution of other Egr-1 regulated genes to TRAIL sensitivity.

The 5′ region of the c-FLIP gene contains an Egr-1 binding site. Given that the Egr-1 binding site is a rare promoter element, and that the mouse c-FLIP promoter also contains an Egr-1 binding site (data not shown), it may be a *bona fide* site and thus indicate a direct regulation of c-FLIP by Egr-1; however, only experimental evidence can confirm it. The c-FLIP promoter also contains a number of AP-1 binding sites and c-Jun is known to be activated by DR4 and DR5. However, inhibition of c-Jun with a dominant-negative construct failed to alter TRAIL sensitivity (data not shown), indicating that c-Jun does not have a major role in regulating c-FLIP expression. Inhibition of Egr-1 affected c-FLIP_S_ expression more than of c-FLIP_L_ probably because of a differential degradation of c-FLIP isoforms. C-FLIP_S_ has been shown to be more prone to ubiquitylation and degradation than c-FLIP_L_. Lysines 192 and 195 are principal ubiquitin acceptors in c-FLIP_S_ but not in c-FLIP_L_ because a 19 amino acid tail, which is specific to c-FLIP_S_ and adjacent to the two target lysines, is required for correct positioning and subsequent ubiquitylation of the target lysines (Poukkula *et al*, 2005).

The considerable level of basal Egr-1 expression in colon carcinoma cells can maintain high c-FLIP expression levels, in particular c-FLIPs, and can thus reduce TRAIL sensitivity. Furthermore, upon DR4/DR5 stimulation Egr-1 becomes induced, which may further increase c-FLIP levels and protect the cells from apoptosis. Knockdown of c-FLIP_S_ confirmed that c-FLIP_S_ regulates DR5-mediated apoptosis more so than of DR4, explaining the specific potentiating effect of DN-Egr-1 of DR5, but not of DR4-induced cell death. This specific effect of c-FLIP_S_ either relates to its differential binding to DR4 *vs* DR5 or to its ability to block type I but not type II apoptosis. It is feasible that despite the presence of c-FLIP_S_ on the DISC, some low level of pro-caspase-8 processing can occur. This would allow the progression of the type II pathway, but would be insufficient to trigger the type I pathway. Inhibition of Egr-1 activity also increased apoptosis induction by other death receptors (TNFRI and Fas) known to be inhibited by c-FLIP. This also indicates that the effect of Egr-1 on c-FLIP expression has a significant biological effect.

In conclusion, this study shows that Egr-1 regulates the expression of c-FLIP in colon carcinoma cells and probably this mechanism contributes to Egr-1-mediated TRAIL resistance. Constitutive Egr-1 expression has been shown to correlate with prostate and gastric tumour aggressiveness and metastasis (Thigpen *et al*, 1996; Kobayashi *et al*, 2002). Recent results at the same time indicate that loss of TRAIL sensitivity of tumour cells is a key step enabling metastasis (Grosse-Wilde *et al*, 2008). The results of our study may shed light on the connection between Egr-1 expression and tumour aggressiveness. Our findings also indicate that the function of DR4 and DR5 is regulated separately intracellularly and the Egr-1 status of a tumour may indicate the sensitivity of the tumour towards death receptor agonist therapeutics.

## Figures and Tables

**Table 1A:** Functional clustering of TRAIL/DR5-variant regulated genes

**Function**	**Number of genes**
*Vesicle and/or protein transport*	4
Rho signalling	2
Post-translational protein modification	3
	
*Transcription/translation*	10
Transcription factors	6
Translational related	4
	
*Cell proliferation/survival pathways*	4
Ras pathway	3
PI3K pathway	1
	
Protein kinases/phosphatase	3
NF-*κ*B inhibitor proteins	2
DNA/RNA helicases	2
Cancer related	8

Abbreviations: DR5=death receptor 5; NF-*κ*B= nuclear factor-*κ*B; TRAIL=tumour necrosis factor-related apoptosis-inducing ligand.

**Table 1B:** TRAIL/DR5-variant regulated genes selected for validation

	**Fold change**			
**Genes**	**TRAIL**	**D269H**	**D269H/E195R**	**Biological function**
*Egr*	2.5	2.1	3.3	Transcription factor
*c-Jun*	1.2	1.6	2.0	Transcription factor
*TEAD1*	−1.6	−1.5	−1.9	Transcription factor
*VDAC3*	−1.5	−1.9	−2.0	Voltage gated anion channel
*NFκB1A/IκBα*	1.3	1.6	2.1	NF-*κ*B inhibitor
*NFκB1Z/IκBζ*	1.8	2.0	2.1	NF-*κ*B inhibitor
*NKD2*	−1.6	−1.3	−1.6	NF*κ*B inhibitor, negative regulator of WNT pathway

Abbreviations: DR5=death receptor 5; Egr=early growth response gene; NKD2= naked cuticle homologue 2; NF-*κ*B= nuclear factor-*κ*B; NFKBIA/I*κ*B*α*=NF-*κ*B inhibitor-*α*/inhibitor-*κ* B-*α* NFKBIZ/I*κ*B*ζ*= NF-*κ* light polypeptide gene enhancer in B-cells inhibitor-*ζ*; TEAD-1=TEA domain family member 1; TRAIL=tumour necrosis factor-related apoptosis-inducing ligand; VDAC3=voltage-dependent anion channel 3.
