# Supplementary figures and images for: Early growth response-1 is a regulator of DR5-induced apoptosis in colon cancer cells
Source: Br J Cancer. 2010 Jan 19;102(4):754–64. doi: 10.1038/sj.bjc.6605545 (PMC2837577; doi:10.1038/sj.bjc.6605545)

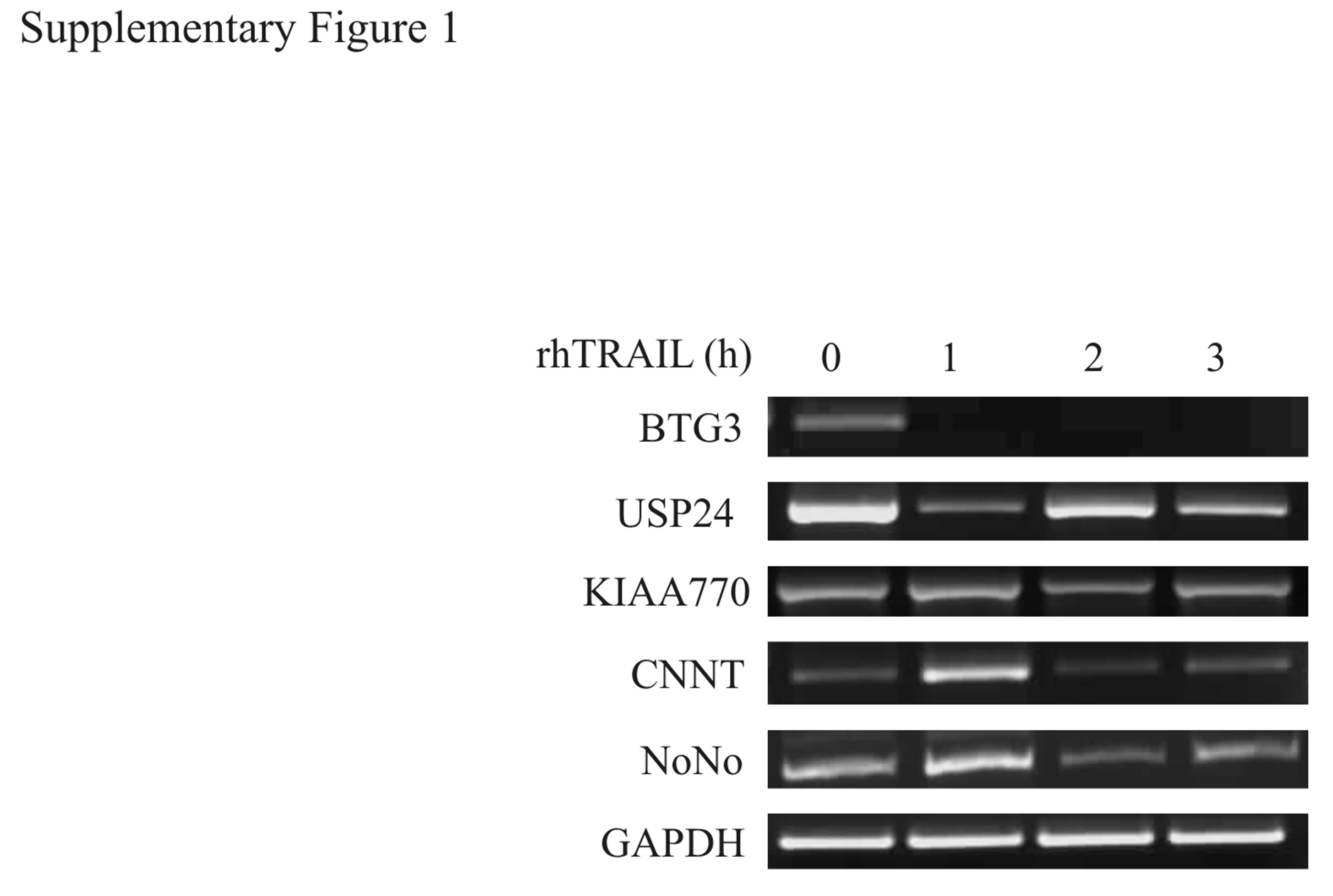

Supplement: Supplementary Figure 1 [file 6605545x1.tif]

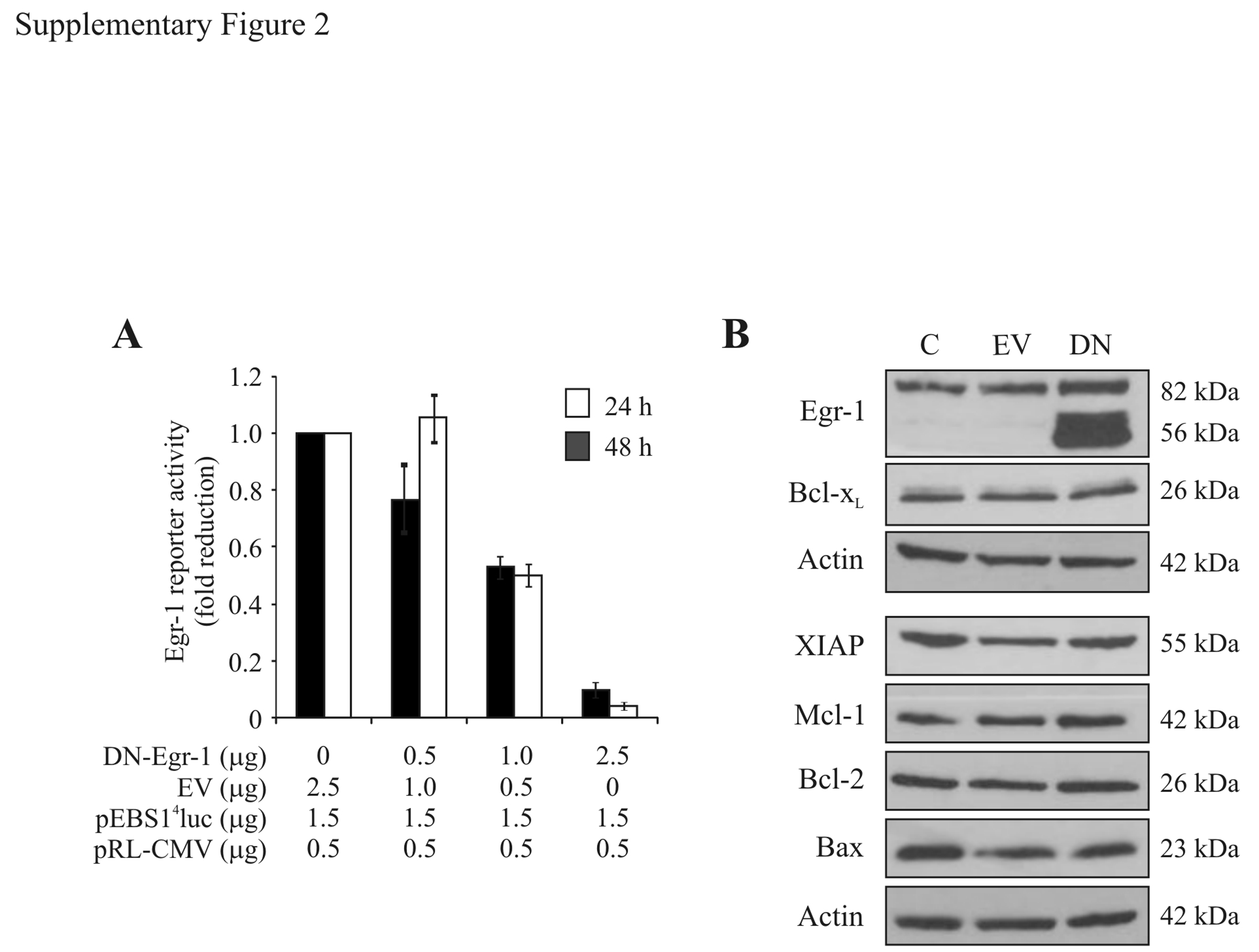

Supplement: Supplementary Figure 2 [file 6605545x2.tif]

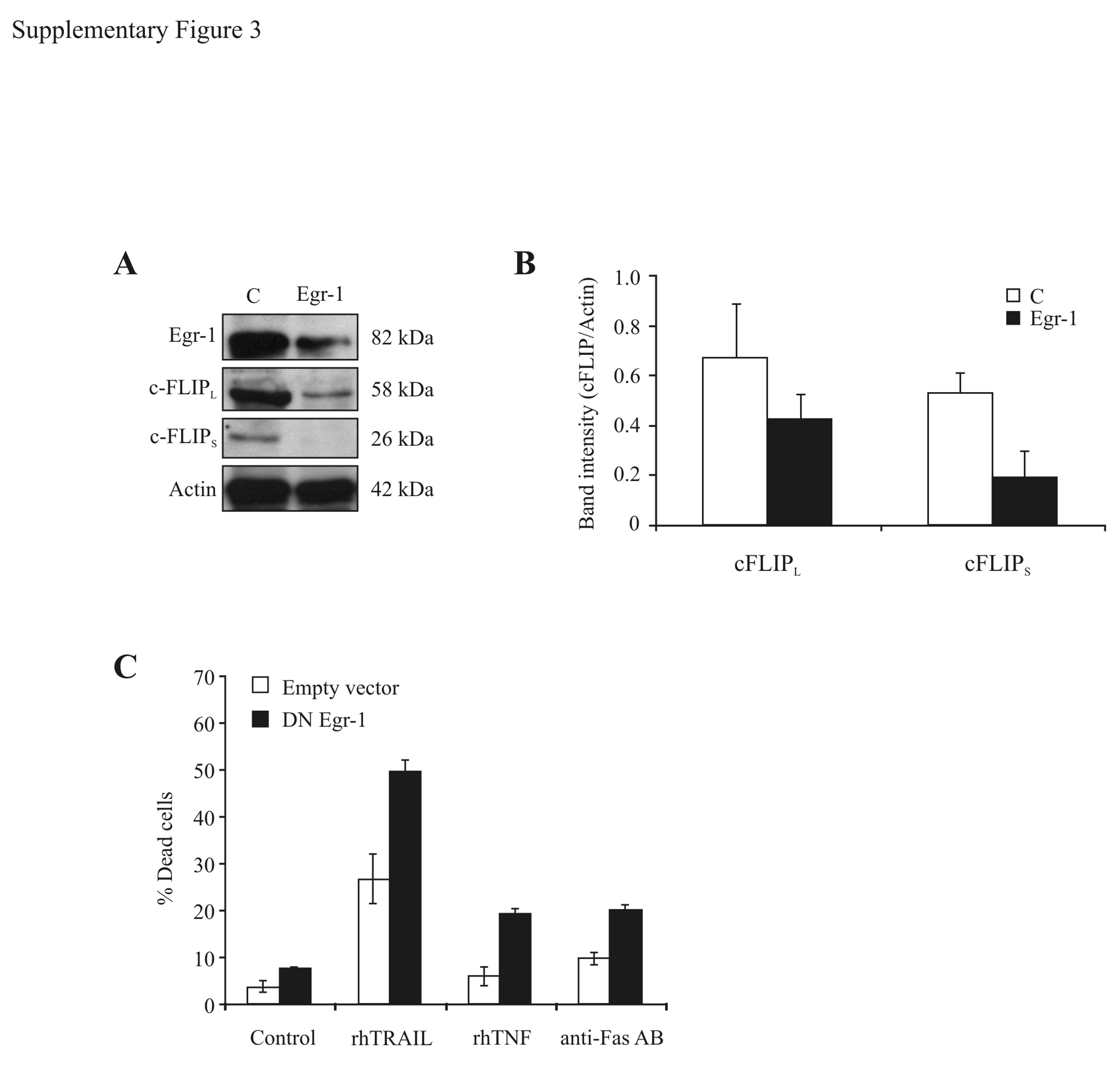

Supplement: Supplementary Figure 3 [file 6605545x3.tif]
