# Supplementary material for: Early growth response-1 is a regulator of DR5-induced apoptosis in colon cancer cells
Source: Br J Cancer. 2010 Jan 19;102(4):754–64. doi: 10.1038/sj.bjc.6605545 (PMC2837577; doi:10.1038/sj.bjc.6605545)
Supplement: Supplementary Figure 4 [file 6605545x4.pdf]

Transcription factor binding sites on the human c-FLIP gene 5' sequence

```
-550   ggctgcagtg agctgtgatg gcgccactgc cttctagcct gggcgacagc  -501
    ···==(7.5231) LBP-1 I00191
    ···====(10.00) GAL4 R00495
                 =====(8.5022) GT-IIBa I00160
                  =====(8.00) LEF-1,TCF-1(P),TCF-1,TCF-1A,TCF-1B,TCF-1C,TCF-1E,TCF-1F,TCF-1G,TCF-2alpha
R02248,R02248,R02248,R02248,R02248,R02248,R02248,R02248,R02248,R02248,R02248
                   ======(12.00) GR R01814
                    ======(10.00) IPF1 R04228
                       === ===(12.00) UCRF-L R01148
                        == ==(8.00) NF-1 R01681
                            ====(8.00) NF-1 R01681
                                          =====(10.00) GAL4 R00495
                                            === ==(7.5231) LBP-1 I00191
                                              = ====(10.00) T-Ag R01372
                                                ====(8.00) E2F+p107 R08845
                                                  =====(10.00) NF-E R00558
                                                   =====(8.5022) GT-IIBa I00160
-500   gcgagaccct gtctcaagaa ggaaaacaaa aaaaaacaaa caaaaacata  -451
       ====(8.00) E2F+p107 R08845
               == ===(10.00) NF-E R00558
                   ========= ==(12.00) delta factor,YY1 R04142,R04142
                         === ===(9.2677) Ttk I00261
                          == ====(12.00) c-Ets-2 R04339
                           = =====(11.00) c-Ets-1 54,c-Ets-1 68,c-Ets-2 58-64,PEA3 R02232,R02232,R02232,R02232,R02232,R02232
                               =======(9.9542) SRY M00148
                                ====== =(14.00) TCF-4E R08644
                                   === ==(9.5346) Hb Q00091
                                    == ===(9.5346) Hb Q00091
                                     = ====(9.5346) Hb Q00091
                                       =====(9.5346) Hb Q00091
                                        =====(9.5346) Hb Q00091
                                         ======== ====(24.00) FOXM1a,FOXM1b,HNF-3alpha,HNF-3B R05102,R05102,R05102,R05102
                                          =======(9.9542) SRY M00148
                                              === ====(9.9542) SRY M00148
                                               == =====(14.00) TCF-4E R08644
                                                  =======(12.00) SEF4 R03650
                                                   =====(9.5346) Hb Q00091
                                                        ====···(8.5507) IHF I00375
-450   cacacaaaaa accacaaagt gtgttcacgt ttgctatgac tcccagacaa  -401
    ···===(8.5507) IHF I00375
```

```
            =====(9.5346) Hb Q00091
             ==== =(9.5346) Hb Q00091
              === ===(7.8406) RC2 I00329
               == ======(12.9708) core-BF I00009
               == ======(13.00) AP-3 (2) R02122
                  ======(10.7734) AML1a M00271
                     ======(12.00) TCF-3 R08594
                      =====(8.00) LEF-1,TCF-1(P),TCF-1,TCF-1A,TCF-1B,TCF-1C,TCF-1E,TCF-1F,TCF-1G,TCF-2alpha
R02248,R02248,R02248,R02248,R02248,R02248,R02248,R02248,R02248,R02248,R02248
                       ====(8.00) Dof2,Dof3,MNB1a,PBF R08440,R08441,R08442,R08443
                       ===== ==(11.2550) CAP/CRP I00372
                                    === ==========(13.00) TAF R02579
                                    === =========(10.00) ABF1 R01923
                                     == ====(10.2026) EBP-45 I00136
                                              ==== ===(14.00) AP-1 R02582
                                              ==== ==(12.00) GCN4 R00829
                                              ====(8.00) AP-1 R00368
                                              ==== ===(10.8420) AP-1 I00270
                                              ==== ==(10.6264) GCN4 I00312
                                                         ====···(12.00) GR R01313
-400   caattgtaaa cactcgcgcc gggcgtggtg gctcacgcct gtaatcccag  -351
    ···==(12.00) GR R01313
             ==== ===(14.00) POU1F1a R02784
                  ======(12.00) Zeste R04956
                  ======(12.00) Zeste R01518
                     ====(8.00) E2F+p107 R08845
                      ======(12.00) ZF5 R01772
                       ===== ==(10.00) GCF R02159
                             ======(8.1888) Sp1 I00295
                             ======(12.00) Sp1 R01702
                             ======(12.00) Sp1 R01498
                                   ==== =(10.00) CAC-binding protein R04295
                                   ==== =(7.6530) CAC-binding I00019
                                     == =====(10.8042) Zta Q00195
                                     == ==(8.00) NF-1 R01681
                                             ===== ==========···(36.00) Crx R09056
                                                    =======(12.0667) Bcd Q00016
                                                    =======(8.1680) Ftz.2 I00243
-350   cactttggga ggccgaggcg ggcggatcac gaggtcaaga gttcgagacc  -301
    ···===(36.00) Crx R09056
         ====(8.00) Dof2,Dof3,MNB1a,PBF R08440,R08441,R08442,R08443
         =====(8.00) LEF-1,TCF-1(P),TCF-1,TCF-1A,TCF-1B,TCF-1C,TCF-1E,TCF-1F,TCF-1G,TCF-2alpha
R02248,R02248,R02248,R02248,R02248,R02248,R02248,R02248,R02248,R02248,R02248
          ======= ==(15.00) LyF-1 R02196
               == ===(8.7167) T-Ag I00215
```

```
                  == ===(10.00) T-Ag R01244
                       ====(8.00) RAF R00256
                         =====(8.7167) T-Ag I00215
                         =====(10.00) T-Ag R01244
                          ===== =(12.00) Sp1 R02860
                           ==== ==(12.00) ER-alpha R04883
                             == =====(14.00) Sp1 R01021
                              = =====(12.00) Sp1 R00017,R00447
                              = =====(12.00) CP1,ETF,Sp1 R01375,R01545,R03039
                              = =====(8.1888) Sp1 I00295
                                       === ===(12.00) HES-1 R04470
                                            ======(9.5247) T3R-alpha I00033
                                            ========(12.5315) COUP I00133
                                            ======(12.00) PPAR-alpha R02233,R02233
                                            ======(12.00) SF-1 R09684
                                             =====(10.00) ER-alpha R01194
                                             ======(7.8406) RC2 I00329
                                             =====(9.5714) ER I00276
                                              ====(8.00) AP-1 R00368
                                                               =···(11.00) abaA R03779
-300   attctggcca acagtgaaac cccgtctcta ctaaacacaa aaattaggcg  -251
    ···=====(11.00) abaA R03779
           ====(8.00) NF-1 R01681
                = ====(8.00) LEF-1,TCF-1(P),TCF-1,TCF-1A,TCF-1B,TCF-1C,TCF-1E,TCF-1F,TCF-1G,TCF-2alpha
R02248,R02248,R02248,R02248,R02248,R02248,R02248,R02248,R02248,R02248,R02248
                        ======= ===(16.00) NF-GMa R02214,R02214
                         ======(11.0190) STE12 I00334
                              = ===(8.00) GAL4 R00496
                                ======(11.00) SIF R02244,R02244
                                ======(10.6822) SIF I00214
                                            =======(14.00) POU1F1a R02784
                                             =======(14.00) HiNF-A R00658
                                                   === ====(12.00) SEF4 R03650
                                                    == ===(9.5346) Hb Q00091
                                                            =====···(12.00) Sp1 R02860
                                                             ====···(12.00) ER-alpha R04883
                                                                =···(8.1888) Sp1 I00295
                                                                =···(12.00) Sp1 R01702
-250   ggcgtgctgg cgcgtgcctg tagtcccagc tactcgggag gctgaggcaa  -201
    ···=(12.00) Sp1 R02860
    ···==(12.00) ER-alpha R04883
    ···=====(8.1888) Sp1 I00295
    ···=====(12.00) Sp1 R01702
              === =(8.00) NF-1 R01681
               == ====(12.00) ZF5 R01772
```

```
                                            ====(8.00) RAF R00256
                                                === ======(12.00) Sp1 R02245,R02245,R02245,R02245,R02245,R02245,R02245,R02245
                                                === ==(10.00) T-Ag R01244
                                                === ==(8.7167) T-Ag I00215
                                                 == ===(10.00) GAL4 R00495
                                                     ========···(10.00) C/EBPbeta,C/EBPdelta R02216,R02216,R02216,R02216
                                                     ========···(11.00) C/EBP,C/EBPalpha R04247,R04247,R04247,R04247,R04247
                                                      =====(10.00) T-Ag R01244
                                                      =====(8.7167) T-Ag I00215
                                                        =====···(9.6976) NF1 I00296
-200   gagaatcgct tgaactagga aggcggaggt tgcagtgagc tgaaattgcg  -151
    ···=(10.00) C/EBPbeta,C/EBPdelta R02216,R02216,R02216,R02216
    ···=(11.00) C/EBP,C/EBPalpha R04247,R04247,R04247,R04247,R04247
    ···=(9.6976) NF1 I00296
            ====(8.00) E2F+p107 R08845
                  ======(12.00) GR R01815
                        ==== ==(10.1632) PEA3 I00212
                        ==== ==(11.00) c-Ets-1 54,c-Ets-1 68,c-Ets-2 58-64,PEA3 R02232,R02232,R02232,R02232,R02232,R02232
                           = =========(11.6096) MalT I00377
                             ======(12.00) Sp1 R02860
                                      = ==========(11.00) EcR R03328
                                                       ======···(10.00) C/EBPbeta,C/EBPdelta R02216,R02216,R02216,R02216
                                                       ======···(11.00) C/EBP,C/EBPalpha R04247,R04247,R04247,R04247,R04247
                                                        =====···(20.00) C/EBPdelta R00239
                                                         ====···(10.00) Sp1 R01021
                                                            =···(8.00) NF-1 R01681
-150   ccactgcact ccagcctggg ccacagagcg agactctgtc tcaaaaaaga  -101
    ···===(10.00) C/EBPbeta,C/EBPdelta R02216,R02216,R02216,R02216
    ···===(11.00) C/EBP,C/EBPalpha R04247,R04247,R04247,R04247,R04247
    ···=====(20.00) C/EBPdelta R00239
    ···=(10.00) Sp1 R01021
    ···===(8.00) NF-1 R01681
           =====(10.00) Sp1 R01021
           ====== =(11.2677) MEP-1 I00078
           ====== =(11.00) MTF-1 R02204
            ===== ====(12.00) TTF-1 R04645,R04645,R04645,R04645,R04645
             ==== ==(12.00) Zeste R04950,R04950
                = ===(8.00) NF-1 R01681
                    =====(10.00) GAL4 R00495
                      =====(7.5231) LBP-1 I00191
                        ==== =(10.00) T-Ag R01372
                           = ===(8.00) NF-1 R01681
                              =====(8.00) LEF-1,TCF-1(P),TCF-1,TCF-1A,TCF-1B,TCF-1C,TCF-1E,TCF-1F,TCF-1G,TCF-2alpha
R02248,R02248,R02248,R02248,R02248,R02248,R02248,R02248,R02248,R02248,R02248
                                ====(10.00) GR R01813
```

```
                                                   === =(8.00) E2F+p107 R08845
                                                          =====(10.00) GR R01813
                                                            =====(10.00) NF-E R00558
                                                                    ========···(12.00) delta factor,YY1 R04142,R04142
                                                                    =====(9.5346) Hb Q00091
                                                                     =====(9.5346) Hb Q00091
                                                                      =====(8.00) LEF-1,TCF-1(P),TCF-1,TCF-1A,TCF-1B,TCF-1C,TCF-1E,TCF-1F,TCF-
1G,TCF-2alpha R02248,R02248,R02248,R02248,R02248,R02248,R02248,R02248,R02248,R02248,R02248
                                                                       ====(8.00) Dof2,Dof3,MNB1a,PBF R08440,R08441,R08442,R08443
                                                                          ==···(9.2677) Ttk I00261
                                                                           =···(12.00) c-Ets-2 R04339
-100   aggaaagaaa gaaagaaaaa aaaaaacact cgcagtgttt actcctaacg  -51
    ···===(12.00) delta factor,YY1 R04142,R04142
    ···====(9.2677) Ttk I00261
    ···=====(12.00) c-Ets-2 R04339
       ======(11.00) c-Ets-1 54,c-Ets-1 68,c-Ets-2 58-64,PEA3 R02232,R02232,R02232,R02232,R02232,R02232
        ======(12.00) NF-1 R00802
        =======(11.5847) GT-IIA I00172
        ======(9.5098) NP-TCII I00400
          ====(8.00) Dof2,Dof3,MNB1a,PBF R08440,R08441,R08442,R08443
            =====(7.8210) HSTF M00028
              === =(8.00) Dof2,Dof3,MNB1a,PBF R08440,R08441,R08442,R08443
                = ====(7.8210) HSTF M00028
                   ====(8.00) Dof2,Dof3,MNB1a,PBF R08440,R08441,R08442,R08443
                     =====(7.8210) HSTF M00028
                       =====(9.5346) Hb Q00091
                        ==== =(9.5346) Hb Q00091
                         === ==(9.5346) Hb Q00091
                          == ===(9.5346) Hb Q00091
                           = ====(9.5346) Hb Q00091
                             =====(9.5346) Hb Q00091
                              =====(9.5346) Hb Q00091
                                   ==== ==(12.00) Zeste R01518
                                   ==== ==(12.00) Zeste R04956
                                      = ===(8.00) E2F+p107 R08845
                                            ====== =(14.00) POU1F1a R02784
                                             ===== ===(10.9837) SGF-1 I00253
                                                == ====(12.00) GCN4 R00647
                                                       ====(8.00) c-Myb R04341
                                                         ===···(10.8441) MCBF I00319
                                                         ===···(10.8441) MCBF I00319
                                                         ===···(12.00) DSC1,MCBF R03655,R03726
-50   cgtggaactt gtgtcgacat ccaccccgg ttactgcata ctcagtcaca  -1
    ···===(10.8441) MCBF I00319
    ···===(10.8441) MCBF I00319
```

···===(12.00) DSC1,MCBF R03655,R03726
====(8.00) NF-1 R01681
===== ===(10.1357) DEP2 I00390
=== ===(12.00) HES-1 R04468
= =====(12.00) GR R03537
=== ==(10.00) GCR1 R03806
= ===(8.00) NF-1 R01681
=====(7.6530) CAC-binding I00019
=====(10.00) CAC-binding protein R04295
=====(10.00) CACCC-binding factor,gammaCAC1,gammaCAC2 R00559,R00559,R01474
====(8.00) GAL4 R00496
= ===(8.00) c-Myb R04341
========= ===(18.00) su(Hw) R02385
======(12.00) EFII R01342
======(12.00) GCN4 R00656
====(8.00) AP-1 R00368
==···(12.00) HES-1 R04468
1 caagccatag caggaaacag cgagcttgca gcctcaccga cgagtctcaa 50
···====(12.00) HES-1 R04468
====(8.00) NF-1 R01681
== ========(13.00) Elk-1 R03591
= =====(9.2677) Ttk I00261
= ====(8.5022) LVc I00085
= ====(10.00) LVc R01644
======(11.00) c-Ets-1 54,c-Ets-1 68,c-Ets-2 58-64,PEA3 R02232,R02232,R02232,R02232,R02232,R02232
=====(8.00) LEF-1,TCF-1(P),TCF-1,TCF-1A,TCF-1B,TCF-1C,TCF-1E,TCF-1F,TCF-1G,TCF-2alpha
R02248,R02248,R02248,R02248,R02248,R02248,R02248,R02248,R02248,R02248,R02248
==== =(8.5022) GT-IIBa I00160
= ===(8.00) E2F+p107 R08845
== =======(18.00) WT1 -KTS,WT1 I -KTS,WT1 I-del2,WT1 I,WT1-del2
R02307,R02307,R02307,R02307,R02307
= ====(10.00) GAL4 R00495
=====(8.7167) T-Ag I00215
=====(10.00) T-Ag R01244
====(8.00) RAF R00256
51 ctaaaaggga ctcccggagc taggggtggg gactcggcct cacacagtga 100
=====(8.00) LEF-1,TCF-1(P),TCF-1,TCF-1A,TCF-1B,TCF-1C,TCF-1E,TCF-1F,TCF-1G,TCF-2alpha
R02248,R02248,R02248,R02248,R02248,R02248,R02248,R02248,R02248,R02248,R02248
====(8.00) Dof2,Dof3,MNB1a,PBF R08440,R08441,R08442,R08443
======== ==(14.00) AP-2,AP-2alpha,AP-2alphaA,AP-2alphaB R05065,R05065,R05065,R05065,R05065,R05065
====(8.00) GAL4 R00496
=====(10.00) CACCC-binding factor,gammaCAC1,gammaCAC2 R00559,R00559,R01474
======= =(13.4484) CACCC-binding_factor Q00019
=======(14.00) PuF R02239
=======(10.6684) CAC-binding_protein Q00018

```
                                                  ======(12.00) Sp1 R08207
                                                  ====== =(14.00) CAC-binding protein R04290
                                                  =====(7.6530) CAC-binding I00019
                                                   ===== =(9.7926) MIG1 I00321
                                                     === =(8.00) GAL4 R00496
                                                            ====(8.00) RAF R00256
                                                               ==== =(10.00) T-Ag R01244
                                                               ==== =(8.7167) T-Ag I00215
                                                                      =====(8.00) LEF-1,TCF-1(P),TCF-1,TCF-1A,TCF-1B,TCF-1C,TCF-1E,TCF-1F,TCF-
1G,TCF-2alpha R02248,R02248,R02248,R02248,R02248,R02248,R02248,R02248,R02248,R02248,R02248
                                                                           ===···(9.7774) zeste I00263
                                                                           ===···(12.00) GCN4 R00830
                                                                           ===···(12.00) Zeste R04945,R04945
101    gtgccggcta ttggactttt gtccagtgac agctgagaca acaaggacca  150
    ···===(9.7774) zeste I00263
    ···===(12.00) GCN4 R00830
    ···===(12.00) Zeste R04945,R04945
              === ====(14.00) gammaCAAT R00563
                = ====(9.5829) CBP/CRF I00135
                = ====(10.00) alpha-CBF,alpha-CP1,alpha-CP2a, alpha-CP2b,alpha-IRP,CDP2,Clox,CP1,CP2,CUTL1,Cutl1,H1TF2,NF-1,NF-
E,SRF,TGGCA-binding protein
R00039,R00510,R00510,R00510,R00532,R00561,R00561,R00562,R00562,R00562,R00562,R00564,R00571,R00572,R00660,R01543,R02848
                = ====(9.8066) CP1 I00066
                = ====(10.00) C/EBPalpha,CBF (1),CBF (2),CBF-A,CBF-B,CCAAT-binding factor,CDF,CRF,CTF,NF-Y',NF-Y
R00231,R00232,R00232,R00232,R00335,R00668,R00669,R00761,R01081,R01445,R01446
                = ====(8.1929) CP2 I00132
                   ====(8.00) NF-1 R01681
                       =====(8.00) LEF-1,TCF-1(P),TCF-1,TCF-1A,TCF-1B,TCF-1C,TCF-1E,TCF-1F,TCF-1G,TCF-2alpha
R02248,R02248,R02248,R02248,R02248,R02248,R02248,R02248,R02248,R02248,R02248
                       ====(8.00) Dof2,Dof3,MNB1a,PBF R08440,R08441,R08442,R08443
                             ========== =(11.00) EcR R03328
                              ====(8.00) NF-1 R01681
                                   ====(8.00) AP-1 R00368
                                    === ==(10.00) NF-E R00558
                                     == =====(11.00) E12,E47 R02139,R02139
                                     == ===(8.5022) GT-IIBa I00160
                                     == =====(11.2677) MyoD I00284
                                     == =====(14.00) GT-IIBalpha,GT-IIBbeta R01418,R01419
                                      = =====(8.6618) Tal-1 I00412
                                      = =====(10.4546) AP-4 Q00009
                                      = =====(8.6618) Tal-1 I00412
                                      = =====(10.4546) AP-4 Q00009
                                             ===== ======(12.00) delta factor,YY1 R04142,R04142
                                               === ===(12.00) GR R01313
                                                       =====(10.00) H4TF-2 R00681
```

```
                                                                =====(7.9248) H4TF2 I00180
151    cgggaggagg tgtaggagag aagcgccgcg aacagcgatc gcccagcacc  200
          =====(10.00) GAL4 R00492
                      =====(10.00) ADR1 R00074
                              ========(11.1281) GCF I00152
                               =======(10.00) GCF R02159
                                 =====(6.4952) T-Ag Q00168
                                  ===== =(12.00) E2F+p107 R08844
                                      = =====(9.5098) LVa I00193
                                      = =====(12.00) LVa R01135
                                        =====(8.00) LEF-1,TCF-1(P),TCF-1,TCF-1A,TCF-1B,TCF-1C,TCF-1E,TCF-1F,TCF-1G,TCF-2alpha
R02248,R02248,R02248,R02248,R02248,R02248,R02248,R02248,R02248,R02248,R02248
                                         =====(8.5022) GT-IIBa I00160
                                            ====(8.00) E2F+p107 R08845
                                                == ==(8.00) E2F+p107 R08845
                                                   =====(10.00) T-Ag R01372
                                                   ========(16.00) AP-2 R01947
201    aagtccgctt ccaggctttc ggtttctttg cctccatctt gggtgcgcct  250
            ===== ==(10.2467) erg I00003
                = ===(8.00) NF-1 R01681
                  =====(7.5231) LBP-1 I00191
                    =====(10.00) GAL4 R00495
                       ====(8.00) Dof2,Dof3,MNB1a,PBF R08440,R08441,R08442,R08443
                          == ==(8.00) RAF R00256
                               =====(7.8210) HSTF M00028
                                  =====(8.00) LEF-1,TCF-1(P),TCF-1,TCF-1A,TCF-1B,TCF-1C,TCF-1E,TCF-1F,TCF-1G,TCF-2alpha
R02248,R02248,R02248,R02248,R02248,R02248,R02248,R02248,R02248,R02248,R02248
                                  ====(8.00) Dof2,Dof3,MNB1a,PBF R08440,R08441,R08442,R08443
                                   ==== ==(12.00) AP-2alpha,AP-2alphaA R08502,R08502
                                      = ====(10.00) T-Ag R01244
                                      = ====(8.7167) T-Ag I00215
                                          ====(8.00) NF-1 R01681
                                           =======(9.7405) R2 I00041
                                           ======(10.00) YY1 R02133
                                           ======= ====(12.00) delta factor,YY1 R04142,R04142
                                                   =====(10.00) CACCC-binding factor,gammaCAC1,gammaCAC2 R00559,R00559,R01474
                                                      =====(10.00) Sp1 R01021
                                                           ==···(12.00) c-Ets-2 R04343
251    tcccggcgtc taggggagcg aaggctgagg tggcagcggc aggagagtcc  300
    ···====(12.00) c-Ets-2 R04343
                    ====(8.00) GAL4 R00496
                     =======(14.00) GAGA factor R02065
                         === =(8.00) E2F+p107 R08845
                              =====(10.00) GAL4 R00495
                                  ===== ====(18.00) ROM1,ROM2 R08458,R08458
```

```
                                     == ===(10.00) CAC-binding protein R04295
                                     == ===(7.6530) CAC-binding I00019
                                        =====(10.00) NF-1/L R01322
                                        ====(8.00) NF-1 R01681
                                            ====== ==(10.9837) BUF I00303
                                             =====(6.4952) T-Ag Q00168
                                                == ===(10.00) LVc R01644
                                                == ===(8.5022) LVc I00085
                                                == ====(9.2677) Ttk I00261
                                                    =====(10.00) ADR1 R00074
301    ggccgcgaca ggacggtacg tgccccgcgc tcgacccccg cgctggcggc  350
        =====(6.4952) T-Ag Q00168
         ======(12.00) E2F+p107 R08844
             ==== =(10.00) NF-E R00558
                             ==========(14.00) GCF R02644
                             =====(10.00) ER-alpha,LF-A1,Sp1 R01171,R04883,R04883
                              =====(10.00) T-Ag R01372
                               ====(8.00) GAL4 R00496
                               ========(10.00) AP-2,AP-2alpha,AP-2alphaA,AP-2alphaB R02121,R02121,R02121,R02121,R02121,R02121
                                            ====== ==(10.00) AP-2,AP-2alpha,AP-2alphaA,AP-2alphaB
R02121,R02121,R02121,R02121,R02121,R02121
                                            ====== ====(12.00) Yi R03154
                                            ====(8.00) GAL4 R00496
                                                      ====(8.00) NF-1 R01681
                                                        =====···(10.00) GCF R02159
                                                        =====(6.4952) T-Ag Q00168
351    gccggagctg tcccagaccc caagccccga cgcccggccc tgagtcagca  400
    ···==(10.00) GCF R02159
             ==== =(8.5022) GT-IIBa I00160
              === ==(10.00) NF-E R00558
               == ====(12.00) GR R03539
                        ==== ==(8.5446) ADR1 M00048
                         === =(8.00) GAL4 R00496
                          == ======(10.00) AP-2,AP-2alpha,AP-2alphaA,AP-2alphaB R02121,R02121,R02121,R02121,R02121,R02121
                                =====(10.00) T-Ag R01372
                                 ====(8.00) GAL4 R00496
                                   ====(8.00) RAF R00256
                                      = =====(8.1888) Sp1 I00295
                                      = =====(12.00) Sp1 R01498
                                      = =====(12.00) Sp1 R01702
                                           =======(10.00) GCF R02159
                                                 = =======(16.00) NF-E2 R02211,R02211,R02211
                                                   ========(16.00) c-Fos,c-Jun R08085,R08085
                                                   ======(12.00) Zeste R04947,R04947
                                                   =======(12.2587) AP-1 I00224
```

```
                                                      =======(11.3365) c-Jun I00010
                                                      =======(11.5040) AP-1 Q00005
                                                      =======(12.0840) AP-1 Q00006
                                                      =======(10.6699) Zta I00413
                                                      =======(12.00) AP-1,YAP1 R02120,R02120,R02120,R02120,R02120
                                                      =======(12.0536) c-Fos I00414
                                                      =======(12.5317) AP-1 Q00004
                                                      =======(14.00) AP-1,c-Fos,c-Jun,Fra-1,v-Jun
R01037,R01037,R01037,R01037,R01037,R01037,R02573
                                                      =======(14.00) AP-1 R02734,R08473,R08489
                                                      =======(13.1213) NF-E2 I00071
                                                      ========(11.8317) Pap1 I00045
                                                      =======(9.3987) v-Jun I00417
                                                       ======(10.6264) GCN4 I00312
                                                       ======(12.00) GCN4 R00829
                                                               =···(9.1555) EFII I00275
                                                               =···(11.00) C/EBP,C/EBPalpha R04247,R04247,R04247,R04247,R04247
                                                               =···(10.00) C/EBPbeta,C/EBPdelta R02216,R02216,R02216,R02216
401    ttgcagcagg ccctgcgccg ccgcgccccg cgtcccaccc cgcgccgctc  450
    ···=====(9.1555) EFII I00275
    ···========(11.00) C/EBP,C/EBPalpha R04247,R04247,R04247,R04247,R04247
    ···========(10.00) C/EBPbeta,C/EBPdelta R02216,R02216,R02216,R02216
            =====(10.00) LVc R01644
            =====(8.5022) LVc I00085
                   =====(10.00) LVc R01644
                   =====(8.5022) LVc I00085
                     =====(10.00) Sp1 R01021
                      ====== =(10.00) GCF R02159
                       ===== ====(18.00) WT1 -KTS,WT1 I -KTS R04866,R04866
                        ==== =(6.4952) T-Ag Q00168
                           = ====(6.4952) T-Ag Q00168
                              ======(12.00) ZF5 R01772
                               =======(10.00) GCF R02159
                                ======= =(11.1281) GCF I00152
                                 =====(10.00) T-Ag R01372
                                  ====(8.00) GAL4 R00496
                                           =======(14.00) PuF R02239
                                           ======(12.00) Sp1 R08207
                                           ======= =(10.00) AP-2,AP-2alpha,AP-2alphaA,AP-2alphaB
R02121,R02121,R02121,R02121,R02121,R02121
                                           =======(10.6684) CAC-binding_protein Q00018
                                            =====(10.00) CAC-binding protein R04295
                                            =====(7.6530) CAC-binding I00019
                                             =====(10.00) CACCC-binding factor,gammaCAC1,gammaCAC2 R00559,R00559,R01474
                                               === =(8.00) GAL4 R00496
```

```
                                                    = ======(10.00) GCF R02159
                                                      ========(11.1281) GCF I00152
                                                      ======(12.00) ZF5 R01772
                                                         =====(6.4952) T-Ag Q00168
                                                              ==···(10.00) GAL4 R00492
451    ctcacagccc tgcgctccgg gaaactgcag gtggccggca gtggccaggg  500
    ···===(10.00) GAL4 R00492
         =====(8.00) LEF-1,TCF-1(P),TCF-1,TCF-1A,TCF-1B,TCF-1C,TCF-1E,TCF-1F,TCF-1G,TCF-2alpha
R02248,R02248,R02248,R02248,R02248,R02248,R02248,R02248,R02248,R02248,R02248
          =====(8.5022) GT-IIBa I00160
               == ===(10.00) LVc R01644
               == ===(8.5022) LVc I00085
                  =====(10.00) Sp1 R01021
                  =======(11.00) MTF-1 R02204
                          == ========== ===(22.00) XPF-1 R02264
                                   ==== =(8.5022) LVc I00085
                                   ==== ===(11.9900) E2A I00130
                                   ==== =(10.00) LVc R01644
                                   ==== ===(11.1757) myogenin Q00111
                                   ==== ===(11.00) E12,E47 R02139,R02139
                                    === ===(9.6721) E12 I00081
                                    === =====(12.0457) E-box-factor I00162
                                    === =====(16.00) IgPE-1 R00849
                                    === =====(16.00) NF-kappaE1,NF-kappaE2 R00897,R00897,R00897,R00897
                                    === ===(8.6618) Tal-1 I00412
                                    === =====(12.2892) NF-InsE3 I00069
                                    === ===(12.00) c-Myc,CAN,USF2 R09477,R09477,R09876
                                      = ====(7.6530) CAC-binding I00019
                                      = ====(10.00) CAC-binding protein R04295
                                         ====(8.00) NF-1 R01681
                                                    ====(8.00) NF-1 R01681
                                                      =======···(10.00) AP-2,AP-2alpha,AP-2alphaA,AP-2alphaB
R02121,R02121,R02121,R02121,R02121,R02121
                                                       =====(7.5231) LBP-1 I00191
                                                          ===···(8.00) GAL4 R00496
                                                            =···(10.00) GCR1 R03806
501    gatggcgggg gcgcttctgg aacctgactc agttttgtag gttccttatc  550
    ···=(10.00) AP-2,AP-2alpha,AP-2alphaA,AP-2alphaB R02121,R02121,R02121,R02121,R02121,R02121
    ···=(8.00) GAL4 R00496
    ···====(10.00) GCR1 R03806
       ========(16.00) NF-D R01219
        ======(12.00) UCRF-L R01148
         ====(8.00) NF-1 R01681
          ======(12.00) ER-alpha R04883
          ======= =(10.00) AP-2,AP-2alpha,AP-2alphaA,AP-2alphaB R02121,R02121,R02121,R02121,R02121,R02121
```

```
       =======(14.00) Sp1 R08677
        ====== ===(16.3682) EGR-1 I00117
        ====== ===(15.3040) WT1+KTS I00016
        ====== ===(18.00) NGFI-C R03332
        ====== ===(18.00) WT1 -KTS,WT1 I -KTS,WT1 I-del2,WT1 I,WT1-del2,WT1 R02262,R02262,R02262,R02262,R02262,R02262
        ====== ===(17.00) Egr-1,EGR3,Krox-20 R02147,R02147,R02147
          ====(8.00) GAL4 R00496
           === ==(10.00) T-Ag R01372
           === ====(10.00) GCF R02159
                      === =(8.00) NF-1 R01681
                             ======= =(11.8317) Pap1 I00045
                             ======= =(16.00) c-Fos,c-Jun R08085,R08085
                              ====== =(14.00) AP-1 R02734,R08473,R08489
                              ====== =(12.5317) AP-1 Q00004
                              ====== =(11.5040) AP-1 Q00005
                              ====== =(12.0536) c-Fos I00414
                              ====== =(10.6699) Zta I00413
                              ====== =(12.0840) AP-1 Q00006
                              ====== =(12.00) AP-1,YAP1 R02120,R02120,R02120,R02120,R02120
                              ======(12.00) GCN4 R00829
                              ====== =(12.2587) AP-1 I00224
                              ====== ==(16.00) NF-E2 R02211,R02211,R02211
                              ====== =(14.00) AP-1,c-Fos,c-Jun,Fra-1,v-Jun R01037,R01037,R01037,R01037,R01037,R01037,R02573
                              ======(10.6264) GCN4 I00312
                              ====== =(9.3987) v-Jun I00417
                              ====== =(11.3365) c-Jun I00010
                              ====== =(13.1213) NF-E2 I00071
                               ===== =(12.00) Zeste R04947,R04947
                                                ======(12.00) c-Ets-2 R04339
                                                    =====···(9.8070) GATA-1 Q00060
                                                    =====···(9.9211) GATA-1 I00109
                                                    =====···(9.8474) GATA-3 I00108
                                                    =====···(12.00) GATA-1,GATA-2 R03884,R03884
                                                    =====(10.00) GAL4,TBP R00494,R03182
                                                    =====···(10.00) GATA-1,GATA-1A,GATA-1B,GATA-2,GATA-3,NF-E1b
R02156,R02156,R02156,R02156,R02157,R02157,R02157,R02157,R02158,R02158,R02158,R02158,R02158
                                                    =====(10.00) ABF2 R03826
                                                     ====···(12.00) NIT2 R02731
                                                     ====···(10.1961) NIT2 Q00133
```

Hit Sense and Strength Colouring Scheme			Colour	Database (section)
Colour	DNA Strand	Secondary Threshold		IMD
=	+	above		TRANSFAC matrix
=	–	above		CBIL matrices
–	+	below		TRANSFAC site
–	–	below		TRANSFAC/CBIL string-matrix

The figure shows the annotated sequence of the human c-FLIP gene 5' region, including 550 nucleotides upstream (-550 to 1) and downstream (1 to 550) of the transcriptional start site (labelled as nucleotide 1). The transcription factor (TF) binding site search was carried out with the Transcription Element Search System (TESS, http://www.cbil.upenn.edu/cgi-bin/tess/tess?RQ=WELCOME). The meaning of the colours is explained in the table above. Best matches of TF binding sites are indicated with double lines, blue colour labels the TF binding sequence found on the sense DNA strand, while red indicates the reverse strand. The value in front of the name of the TF is the La score (Log-likelihood score, higher is better) of the binding site. This score for Egr-1 is the maximum possible. The colours of the TF names indicate (blue, green or purple) what database was the source of the model for a binding site hit. The names of the TFs are followed by the hyperlinked TESS records for the TF binding site. Yellow highlights indicate the GSG motifs (GCG(G/T)GGGCG), the binding site for the Egr TF family.
