# Supplementary material for: Early growth response-1 is a regulator of DR5-induced apoptosis in colon cancer cells
Source: Br J Cancer. 2010 Jan 19;102(4):754–64. doi: 10.1038/sj.bjc.6605545 (PMC2837577; doi:10.1038/sj.bjc.6605545)
Supplement: Supplementary Figure Legends [file 6605545x5.doc]

**Supplementary Figure 1. Temporal induction/repression of known TRAIL-regulated genes.** Colo205 cells were treated with rhTRAIL and total RNA was isolated at the times indicated. Expression of BTG3, USP24, KIAA770, CNNT and NoNo genes is shown detected by RT-PCR analysis. GAPDH expression was analysed as a loading control. The pictures are representatives of two independent experiments.

### Supplementary Figure 2. Overexpression of DN-Egr-1 does not alter multidomain Bcl-2 protein or XIAP expression. (A) Titration of DN-Egr-1. HCT15 cells were transfected with the Egr-1 reporter pEBS1^4^luc (1.5 μg) together with DN-Egr-1 and empty vector (EV) as outlined in the figure. 24 h and 48 h post-transfection Egr-1 activity was monitored by measuring firefly luciferase activity in whole cell lysates. Cells were also transfected with 0.5 μg of Renilla luciferase-expressing plasmid (pRL-CMV) to normalize for transfection efficiency. (B) Effect of DN-Egr-1 on the expression of apoptosis-regulatory proteins. HCT15 cells transiently transfected with *DN*-Egr-1 (DN) or empty vector (EV). 48 h post-transfection expression of DN-Egr-1, Bcl-X_L_, XIAP, Mcl-1, Bcl-2 and Bax have been detected by Western blot analysis. Actin expression was determined to serve as loading control.

**Supplementary Figure 3. Knockdown of Egr-1 reduces c-FLIP expression and enhances TRAIL- and DR5-induced apoptosis.** HCT15 cells were transiently transfected with a Smartpool siRNA mix against Egr-1 (Egr-1) or scrambled siRNA (control siRNA, C). (A) Expression of c-FLIP_L_ and c-FLIP_S_ was examined in whole cell lysates 24 h post-transfection by Western blotting. Actin expression levels were detected as a loading control and Egr-1 expression levels were detected to monitor knockdown efficiency. (B) Densitometric quantification of c-FLIP_L_ and c-FLIP_S_ levels. The graph shows averaged band densities normalised for β-actin levels in whole cell lysates from three independent experiments. (C) Inhibition of Egr-1 increases TNF- and anti-Fas antibody-induced cell death. HCT15 cells were transfected with empty vector (EV) or DN-Egr-1 before treatment with rhTRAIL (100 ng/ml), rhTNF (60 ng/ml) or agonistic anti-Fas antibody (100 nM) for 4 h. Induction of apoptosis was determined on cytospins stained with hematoxylin-eosin by counting 300 cells/slide.

**Supplementary Figure 4. Annotated sequence of the 5’ region of the human c-FLIP gene**. The sequence of 550 nucleotides upstream (-550 to 1) and downstream (1 to 550) of the transcriptional start site (labelled as nucleotide 1 on the figure) has been analysed for potential transcription factor (TF) binding sites. The TF binding site search was carried out with the Transcription Element Search System web interface (TESS, http://www.cbil.upenn.edu/cgi-bin/tess/tess?RQ=WELCOME). The meaning of the colours is explained in the table at the end of the annotated sequence. Best matches of TF binding sites are indicated with double lines, Blue colour labels the TF binding sequence found on the sense DNA strand, while red indicates the reverse strand. The value in front of the name of the TF is the La score (Log-likelihood score, higher is better) of the binding site. This score for Egr-1 is the maximum value, possible. The colours of the TF names indicate (blue, green or purple) what database was the source of the model for the binding site hit. The names of the TFs are followed by the hyperlinked TESS records for the TF binding site. Yellow highlights indicate the GSG motifs (GCG(G/T)GGGCG), the binding site for the Egr TF family.
