# Supplementary material for: Early growth response-1 is a regulator of DR5-induced apoptosis in colon cancer cells
Source: Br J Cancer. 2010 Jan 19;102(4):754–64. doi: 10.1038/sj.bjc.6605545 (PMC2837577; doi:10.1038/sj.bjc.6605545)
Supplement: Supplementary Table 1 [file 6605545x6.doc]

**Supplementary Table 1. Genes regulated by rhTRAIL, D269H and D269H/E195R**

| **Gene Name** | **Fold Change - TRAIL** | **Fold Change - D269H** | **Fold Change - D269H/E195R** | **Genbank** | **P-value - TRAIL** | **P-value - D269H** | **P-value - D269H/E195R** |
| --- | --- | --- | --- | --- | --- | --- | --- |
| EGR1 | 2.48 | 2.14 | 3.33 | NM_001964 | 0.0001 | 0.0025 | 0.0000 |
| EGR1 | 2.30 | 2.43 | 3.50 | AI459194 | 0.0004 | 0.0000 | 0.0000 |
| NFKBIZ | 1.84 | 2.04 | 2.00 | BE646573 | 0.0003 | 0.0001 | 0.0001 |
| CDC42EP1 | 1.59 | 2.26 | 1.85 | NM_007061 | 0.0037 | 0.0010 | 0.0001 |
| SQSTM1 | 1.42 | 1.73 | 2.50 | AW293441 | 0.0059 | 0.0003 | 0.0036 |
|  | 1.42 | 1.86 | 4.25 | AI825833 | 0.0055 | 0.0001 | 0.0031 |
| PTPRO; PTPU2; GLEPP1 | 1.36 | 1.77 | 2.03 | U20489 | 0.0420 | 0.0001 | 0.0001 |
| DKFZp313A2432 | 1.31 | -1.08 | -1.16 | AI742358 | 0.3109 | 0.4225 | 0.0719 |
| NFKBIA | 1.28 | 1.64 | 2.13 | AI078167 | 0.0253 | 0.0009 | 0.0008 |
| ARL11 | 1.28 | 1.58 | 2.14 | NM_138450 | 0.1981 | 0.0672 | 0.0002 |
| JUN | 1.20 | 1.63 | 1.99 | NM_002228 | 0.0839 | 0.0006 | 0.0048 |
| PTP9Q22 | 1.19 | -1.24 | -1.31 | AA651920 | 0.1820 | 0.0960 | 0.0663 |
|  | 1.16 | 1.29 | 2.25 | NM_013307 | 0.1777 | 0.3273 | 0.0179 |
| ICK | 1.15 | 1.25 | 2.04 | NM_014920 | 0.3574 | 0.1086 | 0.0023 |
| RARS | 1.14 | 1.02 | -2.45 | NM_002887 | 0.4161 | 0.9184 | 0.0150 |
| RNU2 | 1.07 | 1.06 | 2.00 | BC003629 | 0.5611 | 0.6209 | 0.0304 |
|  | 1.05 | 1.39 | 2.35 | BC001131 | 0.7530 | 0.0079 | 0.0024 |
| FLJ10525 | 1.02 | -1.07 | -1.26 | BF114679 | 0.8529 | 0.4992 | 0.0515 |
| C22orf2 | 1.01 | -1.03 | -1.92 | AI808192 | 0.9354 | 0.6716 | 0.0050 |
|  | -1.01 | -1.28 | -2.04 | BU623906 | 0.9583 | 0.1138 | 0.0059 |
| HSPC039; PRO2309 | -1.02 | -1.35 | -2.04 | AF119875 | 0.8696 | 0.1552 | 0.0023 |
| LCHN | -1.02 | 1.06 | -1.49 | AA029331 | 0.8245 | 0.5801 | 0.0745 |
| PLSCR4 | -1.03 | -1.33 | -2.21 | NM_020353 | 0.6904 | 0.0650 | 0.0047 |
| LOC285927 | -1.04 | -1.54 | -2.39 | BC044242 | 0.6736 | 0.1948 | 0.0021 |
| RAB2 | -1.05 | -1.42 | -2.04 | AI189609 | 0.7001 | 0.2043 | 0.0045 |
|  | -1.06 | 1.06 | 2.04 | AU146717 | 0.6795 | 0.5366 | 0.0000 |
|  | -1.06 | -1.75 | -2.35 | AF285120 | 0.6278 | 0.2175 | 0.0040 |
| TRIM2 | -1.09 | -1.26 | -3.25 | AA149745 | 0.6273 | 0.2262 | 0.0026 |
| LOC134492 | -1.10 | -1.57 | -2.38 | AI291200 | 0.3999 | 0.0795 | 0.0057 |
|  | -1.11 | -1.21 | -3.96 | AFFX-HUMRGE/M10098_5 | 0.2958 | 0.4570 | 0.0927 |

| **Gene Name** | **Fold Change - TRAIL** | **Fold Change - D269H** | **Fold Change - D269H/E195R** | **Genbank** | **P-value - TRAIL** | **P-value - D269H** | **P-value - D269H/E195R** |
| --- | --- | --- | --- | --- | --- | --- | --- |
| FLJ20533 | -1.12 | -1.44 | -2.09 | AA886902 | 0.2885 | 0.1270 | 0.0048 |
| PRO1073 | -1.12 | -1.34 | -1.94 | AL037917 | 0.5808 | 0.0282 | 0.0276 |
|  | -1.13 | -1.04 | -2.38 | NM_024084 | 0.3241 | 0.7102 | 0.0035 |
| ALG5 | -1.13 | -1.82 | -2.37 | AF102850 | 0.1986 | 0.0791 | 0.0030 |
|  | -1.14 | -1.53 | -2.26 | AA134418 | 0.3862 | 0.0398 | 0.0030 |
| DHX29 | -1.14 | -1.49 | -2.03 | AL079292 | 0.1868 | 0.1626 | 0.0050 |
| G3BP | -1.14 | -1.46 | -2.13 | BE673925 | 0.2913 | 0.0239 | 0.0066 |
| TRIM33 | -1.15 | -1.31 | -1.65 | AF220137 | 0.2039 | 0.0757 | 0.0098 |
| TRIM2 | -1.15 | -1.18 | -2.27 | AW071795 | 0.5462 | 0.0981 | 0.0039 |
| TTC17 | -1.15 | -1.20 | -2.44 | AK026217 | 0.4035 | 0.1718 | 0.0021 |
| CDC10 | -1.18 | -1.74 | -2.31 | BF855173 | 0.2737 | 0.0233 | 0.0087 |
| PRO1073 | -1.19 | -1.38 | -1.90 | AF132202 | 0.3988 | 0.0447 | 0.0151 |
| PRO1073 | -1.20 | -1.32 | -1.90 | AF113016 | 0.3239 | 0.2687 | 0.0145 |
| NOL7; RARG-1 | -1.21 | -1.65 | -2.08 | AF130102 | 0.2136 | 0.2435 | 0.0132 |
|  | -1.21 | -1.27 | -2.17 | AF339768 | 0.2886 | 0.1949 | 0.0038 |
| NCKAP1 | -1.23 | -2.03 | -2.29 | AK001291 | 0.1679 | 0.0411 | 0.0060 |
| MGC33371 | -1.25 | -2.52 | -2.01 | NM_144664 | 0.1150 | 0.0305 | 0.0048 |
| ATP6V0E | -1.28 | -1.85 | -2.26 | NM_003945 | 0.0542 | 0.0645 | 0.0021 |
| MID1 | -1.28 | -1.29 | -1.44 | AF041209 | 0.1323 | 0.1111 | 0.0185 |
| TEAD1 | -1.28 | -1.21 | -1.90 | AW771935 | 0.1801 | 0.0890 | 0.0104 |
|  | -1.28 | -1.01 | -1.85 | AC005591 | 0.1822 | 0.9453 | 0.0066 |
| KIAA0779 | -1.28 | -1.19 | -2.49 | AB018322 | 0.0767 | 0.1452 | 0.0073 |
| VDR | -1.29 | -1.24 | -2.24 | AA772285 | 0.0787 | 0.0512 | 0.0026 |
|  | -1.30 | -1.61 | -2.23 | NM_014941 | 0.0553 | 0.0101 | 0.0047 |
| LOC56851 | -1.30 | -1.48 | -2.03 | AA149655 | 0.0844 | 0.1956 | 0.0046 |
| CLDN2 | -1.36 | -1.77 | -2.87 | AF177340 | 0.2658 | 0.0666 | 0.0031 |
| ZNF3 | -1.37 | -1.27 | -2.17 | AI752257 | 0.1733 | 0.2113 | 0.0048 |
| TRIM2 | -1.39 | -1.16 | -2.83 | BC005016 | 0.3908 | 0.2651 | 0.0026 |
| AFAP | -1.44 | -1.29 | -2.11 | BC043614 | 0.0669 | 0.0525 | 0.0553 |
|  | -1.45 | -1.90 | -1.98 | BC002456 | 0.0468 | 0.0662 | 0.0112 |
| NAPE-PLD | -1.46 | -2.14 | -1.37 | W01715 | 0.2362 | 0.0207 | 0.0501 |
| CYB561 | -1.53 | -1.33 | -2.06 | NM_001915 | 0.0760 | 0.0924 | 0.0043 |
| dJ406P24.1 | -1.53 | -1.33 | -2.00 | AL138831 | 0.0263 | 0.3582 | 0.0041 |
| NKD2 | -1.55 | -1.25 | -1.55 | BC004940 | 0.0722 | 0.0669 | 0.0096 |
| TEAD1 | -1.59 | -1.50 | -1.85 | NM_021961 | 0.1079 | 0.0216 | 0.0419 |
|  | -1.60 | -1.36 | -2.03 | BE138647 | 0.0606 | 0.2197 | 0.0234 |
| RNF146 | -1.64 | -1.20 | -2.26 | NM_030963 | 0.1412 | 0.3710 | 0.0307 |
|  | -2.25 | -2.75 | -1.26 | AL024509 | 0.1250 | 0.0089 | 0.0622 |
